# Federated learning with swarm intelligence for efficient and secure medical image analysis

**DOI:** 10.1038/s41598-026-50882-8

**Published:** 2026-05-11

**Authors:** M. A. SayedElahl, R. M. Farouk, Abd Elmounem Ali, Elham Ahmed

**Affiliations:** 1 Department of Computer Science, Faculty of Computers and Information, Damanhour University, Damanhour, Egypt; 2 Department of Mathematics, Faculty of Science, Zagazig University, Zagazig, Egypt

**Keywords:** Federated learning, Medical image analysis, Swarm intelligence, Privacy-preserving AI, Deep learning, Cancer imaging, Classification and taxonomy, Computational models, Image processing, Machine learning

## Abstract

Collaborative learning in healthcare faces challenges, including strict regulations and fragmented data. This research introduces a federated learning framework that employs swarm intelligence to augment communication and enhance the analysis of medical images. The method optimizes hyperparameters, selects features, and assigns aggregation weights to federated clients simultaneously by combining Particle Swarm Optimization (PSO) and the Firefly Algorithm (FA) with deep Convolutional Neural Networks (CNNs). The framework was tested on three medical datasets: COVID-19 chest X-rays (5,856 images), monkeypox skin images (569 images), and breast cancer mammograms (320 images). These datasets were shared among four fake healthcare institutions. It strives to strike a balance between privacy, communication costs, and classification accuracy. The results showed that the test was 96.71% accurate in detecting COVID-19, 96.06% accurate in classifying monkeypox, and 97.0% accurate in diagnosing breast cancer. The framework was able to handle noise and attacks from individuals who sought to disrupt it, which reduced communication rounds by 25–30%. A privacy-utility analysis revealed that there were acceptable trade-offs, with accuracy remaining above 94%. This study employs robust privacy measures and statistical validation. It also shows how to use medical AI in smaller healthcare settings without putting patients’ privacy at risk.

## Introduction

Data is fundamental to any organization or business, encompassing letters, numbers, or symbols that require processing to yield accurate and actionable information. The ability of data to identify trends and influence future decisions underscores its significance. This has led to the emergence of information and data security as a distinct field, focused on safeguarding public and private data with high confidentiality. Privacy concerns, both natural and regulatory, have prompted the development of robust laws to protect privacy in digital and real-world contexts. In the healthcare sector, managing and securing vast amounts of sensitive big data poses significant challenges. Healthcare data, originating from patient registrations, laboratory tests, and medical procedures, is often localized within outpatient clinics and public hospitals. Each facility maintains electronic health records, which include detailed patient information and prevalent disease data. Strict privacy regulations and the size of these datasets make data sharing between clinics and hospitals a complex task ^1^. Machine learning (ML) has seen remarkable advancements in recent years, with applications spanning healthcare, computer vision, and wireless communications. ML models rely on large, diverse datasets to achieve high predictive accuracy. Linking multiple data sources is essential to create comprehensive datasets while employing advanced methods to detect attacks with minimal human intervention. However, centralized data aggregation, historically used for model training, raises significant privacy concerns and limits access to sensitive data. Federated Learning (FL), introduced by Google in 2016, provides a decentralized solution by enabling collaborative model training across institutions without transferring sensitive data to a central server ^2^. Despite its promise, FL faces challenges in communication efficiency, feature selection, and model optimization when dealing with diverse datasets. This study proposes an optimized FL framework tailored for medical image analysis to address these challenges. Specifically, the study aims to enhance classification accuracy and computational efficiency while preserving data privacy. The research objectives include:

- Developing an FL framework incorporating Convolutional Neural Networks (CNNs) for effective feature extraction across diverse medical datasets.
- Leveraging Particle Swarm Optimization (PSO) and Firefly Algorithm (FA) to optimize feature selection, reduce communication overhead, and improve model performance.
- Evaluating the proposed framework on datasets for COVID-19, Monkeypox, and Breast Cancer to validate its scalability, accuracy, and privacy-preserving capabilities.

Federated learning, or collaborative learning, enables training machine learning models without moving data from client devices to central servers. It enhances privacy by training models locally and combining local updates to create a global model ^3^. Federated learning is essential for protecting privacy, securing data, and accessing diverse datasets. It allows models to be trained on sensitive data like healthcare or financial information without compromising privacy. Due to advancements in the medical field, federated learning holds promise for improving healthcare by enabling communication between various parties without violating privacy. This is crucial for addressing health information challenges and improving healthcare services globally. Federated learning allows access to a larger range of data sources, improving disease diagnosis and classification in the medical field. This approach aids healthcare professionals in leveraging existing data while maintaining security and privacy, benefiting doctors, patients, and researchers. Federated learning is especially advantageous for handling variables from big data sources, helping predict future healthcare decisions, including disease diagnosis, intensive care stay duration, mortality rates, and treatment options. Today, privacy-protected data processing is a key research area, but feature selection with privacy protection remains underexplored. Existing methods for integrating privacy protection into feature selection are either passive, applying an anonymity mechanism to selected features post-selection, or active, which incorporates privacy mechanisms during the feature selection process. Active approaches, such as privacy-aware filtering and wrapping systems, are designed to maintain classification accuracy while addressing privacy concerns. However, these methods are suited for centrally stored data and may not be applicable when data is held separately by different participants who cannot share it. Metaheuristic algorithms have gained widespread use in solving feature selection problems. SayedElahl et al. ^4^ developed a novel edge detection filter based on fractional order Legendre-Laguerre functions, showcasing its application in optimization tasks within intelligent systems. Similarly, SayedElahl and Farouk ^5^ proposed a robust segmentation model for unshaped microarray spots using fractal transformation, demonstrating the potential of advanced optimization techniques in segmentation tasks. Evolutionary computation is popular for solving feature selection problems due to its global search capabilities ^6^. Swarm intelligence methods have increasingly been applied in various fields such as optimization, robot control, and telecommunication network management. Notable techniques include Ant Colony Optimization ^7^, Particle Swarm Optimization ^8^, and Artificial Bee Colony ^9^, alongside more recent algorithms like the Firefly Algorithm ^10^, cuckoo-search ^11^, and the bat algorithm ^12^. Swarm intelligence algorithms, such as Particle Swarm Optimization, are preferred due to their fast convergence and simplicity, making them ideal for addressing feature selection problems in federated learning under privacy protection. This paper explores Particle Swarm Optimization to construct a robust feature selection model for optimal classification results ^13^. The Firefly Algorithm has also proven effective in optimizing various fields, including healthcare ^14^, with applications in medical imaging highlighting its simple yet efficient problem-solving abilities.

### Contributions and novelty

This research introduces a novel framework that addresses the common challenges in FL. It makes significant contributions that improve the privacy and efficiency of medical image analysis, through the following main techniques:

- *Dual Swarm Intelligence Integration*
  - *Innovation* First-of-its-kind hybridization of PSO and FA within FL architecture for medical imaging applications.
  - *Impact* This approach improves accuracy by 4-7% compared to traditional methods while also lowering communication costs by 25%. It achieves this by optimizing hyperparameters and selecting the best features at the same time.
  - *Novelty* Most current FL frameworks use fixed settings. In contrast, our adaptive optimization changes over time. It adjusts based on the characteristics of local data and feedback on overall performance.
- *Multi-Domain Medical Validation Datasets*
  - *Innovation* Comprehensive evaluation across three different areas of medical imaging, focusing on new health threats.
  - *Impact* Demonstrates universal applicability with accuracies exceeding 96% across COVID-19, Monkeypox, and breast cancer datasets, establishing cross-domain generalizability.
  - *Novelty* First federated learning study to systematically address medical image classification while simultaneously validating on established and emerging medical conditions, bridging the gap between pandemic preparedness and routine diagnostics.
- *Edge-Optimized Federated Architecture*
  - *Innovation* A lightweight framework for FL designed for limited-resource environments and mobile health care applications.
  - *Impact* This technology enables real-time health checks in remote areas with minimal computer requirements. It makes AI-based medical analysis accessible to everyone.
  - *Novelty* This research aims to bridge the gap in current FL by emphasizing practical applications rather than solely theoretical advancements. It seeks to make FL practical in low-resource healthcare settings.
- *Privacy-First Security Protocol*
  - *Innovation* Combining various methods to protect privacy is essential for maintaining the integrity of medical data. Key techniques include differential privacy with secure data collection.
  - *Impact* This framework provides highly accurate diagnoses while ensuring strong privacy protection. It meets international healthcare regulations.
  - *Novelty* Establishes the comprehensive privacy framework for federated medical imaging that balances diagnostic utility with patient confidentiality requirements.
- *Temporal Intelligence Foundation*
  - *Innovation* Framework architecture designed to accommodate future video-based medical analysis and temporal pattern recognition.
  - *Impact* This system establishes a robust foundation for monitoring diseases and understanding their progression more flexibly, extending beyond merely classifying images.
  - *Novelty* Anticipates the evolution toward temporal medical AI by establishing compatible federated learning infrastructure for longitudinal patient monitoring.

### Distinctive advantages over existing approaches

Current research in FL primarily focuses on improving algorithms, but often fails in the real-world challenges of deployment. Our framework prioritizes scalability and firm performance in healthcare settings. It optimizes the use of standard clinical hardware and ensures that privacy measures meet the highest healthcare standards. We also promote healthcare equity by quickly adapting to emerging diseases and effectively using resources in low-infrastructure environments. Our goal is to provide accessible AI diagnostics for underserved populations. Unlike traditional FL, our adaptive approach selects algorithms based on medical imaging characteristics. It adjusts parameters in real time and improves outcomes using feedback from collective intelligence.

## Literature review

FL has emerged as a transformative paradigm for medical image analysis, enabling decentralized model training across institutions without compromising patient privacy. This section reviews recent advances in FL applied to three clinical domains: COVID-19 detection, monkeypox diagnosis, and breast cancer screening, with emphasis on studies that integrate optimization techniques, particularly swarm intelligence, and edge computing architectures. A comprehensive summary of representative works is provided in Table 1.

### Mobile edge computing and 5G integration in healthcare

The convergence of Mobile Edge Computing (MEC) and 5G networks has addressed critical latency and bandwidth constraints in healthcare applications. Ghadi et al. ^15^ demonstrated that MEC reduces decision-making latency and improves quality of care in IoT-based health systems. Subsequent studies have shown that MEC-assisted FL achieves energy-efficient, privacy-preserved healthcare services through optimized resource allocation and differential privacy mechanisms ^16^. Systems such as FLERMS-IoT and FedHealthFog further validate the feasibility of FL deployments in resource-constrained environments, achieving over 90% efficiency in high-density scenarios ^17,18^. These developments enable real-time diagnostic applications by offloading computational tasks from medical IoT devices to edge servers while maintaining data locality.

### COVID-19 detection using federated learning

Early applications of FL for COVID-19 detection focused on chest X-ray (CXR) and CT imaging. Alam et al. ^19^ demonstrated FL viability on low-power edge devices, while FedSGDCOVID achieved 95.32% accuracy using the FedAvg algorithm ^20^. A multinational study reported 95.66% accuracy across diverse CT datasets, confirming FL’s robustness to distribution shifts ^21^. Durga and Poovammal ^22^ integrated capsule networks with blockchain, attaining 99.59% accuracy through secure model aggregation. Recent work by Kandati et al. ^23^ incorporated Particle Swarm Optimization (PSO) within FL, achieving 96.15% accuracy while reducing communication overhead by 25%. These studies underscore the synergy between optimization algorithms and FL for respiratory pathology classification.

### Monkeypox detection with limited data

The 2022 monkeypox outbreak highlighted the need for rapid diagnostic tools in emerging disease scenarios. Kumar et al. ^24^ evaluated CNN architectures combined with classical classifiers, reporting 91.11% accuracy with Naïve Bayes. Lightweight architectures proved effective: MobileNetV2 achieved 99% accuracy, suitable for edge deployment ^25^. Abdelhamid et al. ^26^ applied PSO-BER optimization to feature selection, attaining 95.2% accuracy. Kundu et al. ^27^ demonstrated that Vision Transformers (ViT-B32) within FL frameworks reach 97.90% accuracy on GAN-augmented datasets, illustrating the potential of combining advanced architectures with distributed learning for rare diseases.

### Breast cancer diagnosis and advanced imaging

Breast cancer detection has benefited from hybrid deep learning and 3D imaging techniques. Islam et al. ^28^ established baselines using SVM (98.57%) and ANN (97.14%). Recent transformer-inspired models, such as EfficientNetB0+ResNet50 ensembles, achieve 94% accuracy on histopathology images with strong correlation metrics ^29^. PSO has been extensively employed for feature selection, boosting classifier performance across SVM, Naïve Bayes, and decision trees ^30,31^. In federated settings, Kumbhare et al. ^32^ combined DenseNet with Hybrid Dragon-Rider Optimization (HDRO) to reach 95% accuracy on mammograms. Advanced segmentation networks (UltraSegNet, HMA-Net) further enhance tumor localization in ultrasound and mammography ^33,34^. Federated ensemble systems like FDEIoL have demonstrated 99.24% accuracy across multi-modal imaging, showcasing the maturity of FL for oncological applications ^35^.

### Synthesis and research gap

The literature reveals a clear progression from basic FL implementations to sophisticated hybrid systems incorporating edge computing, transformer architectures, and multi-modal imaging. However, most existing approaches treat optimization as an ancillary component rather than an integral part of the federated pipeline. Studies that do employ metaheuristics, such as PSO, typically apply them to isolated tasks (e.g., feature selection on centrally pooled data) rather than embedding them within the federated learning loop. Furthermore, simultaneous optimization of multiple objectives—accuracy, communication cost, convergence speed, and privacy—remains underexplored. The present work addresses this gap by integrating PSO and Firefly Algorithm at both client and server levels, enabling adaptive hyperparameter tuning, feature selection, and aggregation weight optimization in a unified framework validated across three distinct medical imaging domains.

**Table 1:** Summary of Recent Federated Learning Approaches in Medical Image Analysis.

Study	Technique/Method	Dataset	Performance	Year
*COVID-19 Detection*				
Ho et al. ^20^	FedSGDCOVID + SGD	Chest X-ray	95.32% Accuracy	2022
Dou et al. ^21^	Multinational FL	CT Images	95.66% Accuracy	2021
Durga et al. ^22^	FLED-Block + Blockchain	CXR Images	99.59% Accuracy	2022
Kandati et al. ^23^	FL-PSO Optimization	CXR Dataset	96.15% Accuracy	2023
*Monkeypox Detection*				
Kumar et al. ^24^	CNN + Naïve Bayes	Monkeypox Images	91.11% Accuracy	2022
Jaradat et al. ^25^	MobileNetV2	Monkeypox Dataset	99% Accuracy	2023
Kundu et al. ^27^	FL + ViT-B32	Monkeypox Images	97.90% Accuracy	2024
Abdelhamid et al. ^26^	PSO-BER Algorithm	Skin Lesion Images	95.2% Accuracy	2022
*Breast Cancer Diagnosis*				
Shahzad et al. ^29^	EfficientNetB0 + ResNet50	Histopathology Images	94% Accuracy	2025
Kumbhare et al. ^32^	FL + HDRO Optimization	Mammogram Images	95% Accuracy	2023
Advanced FL ^35^	FDEIoL Ensemble	MRI + CXR Images	99.24% Accuracy	2024
Hybrid 3D ^36^	3D Imaging + Segmentation	Mammography + US	96.5% Accuracy	2024
UltraSegNet ^33^	Hybrid DL Framework	BUSI Dataset	95.8% Dice Score	2025
*Optimization and Privacy*				
Agustian et al. ^30^	PSO Feature Selection	Wisconsin Dataset	92.26% Accuracy	2020
Afolayan et al. ^31^	PSO + Multiple Classifiers	Breast Cancer Data	94.8% Accuracy	2022
BFL-MND ^37^	Blockchain + FL	PhysioNet Dataset	95% Accuracy	2024
HierSFL ^38^	Split FL + Privacy	Mobile Edge Data	93.5% Accuracy	2024

## Methodology

This investigation establishes a privacy-preserving collaborative framework for medical image analysis across distributed healthcare institutions. The framework addresses critical challenges in federated learning by integrating swarm intelligence optimization algorithms with traditional deep learning architectures. All datasets employed in this research are publicly accessible and were obtained according to the terms established by their respective providers. Since this study utilized existing datasets without collecting new human subjects data, institutional review board approval was not required.

### Experimental framework architecture

**Fig. 1:**
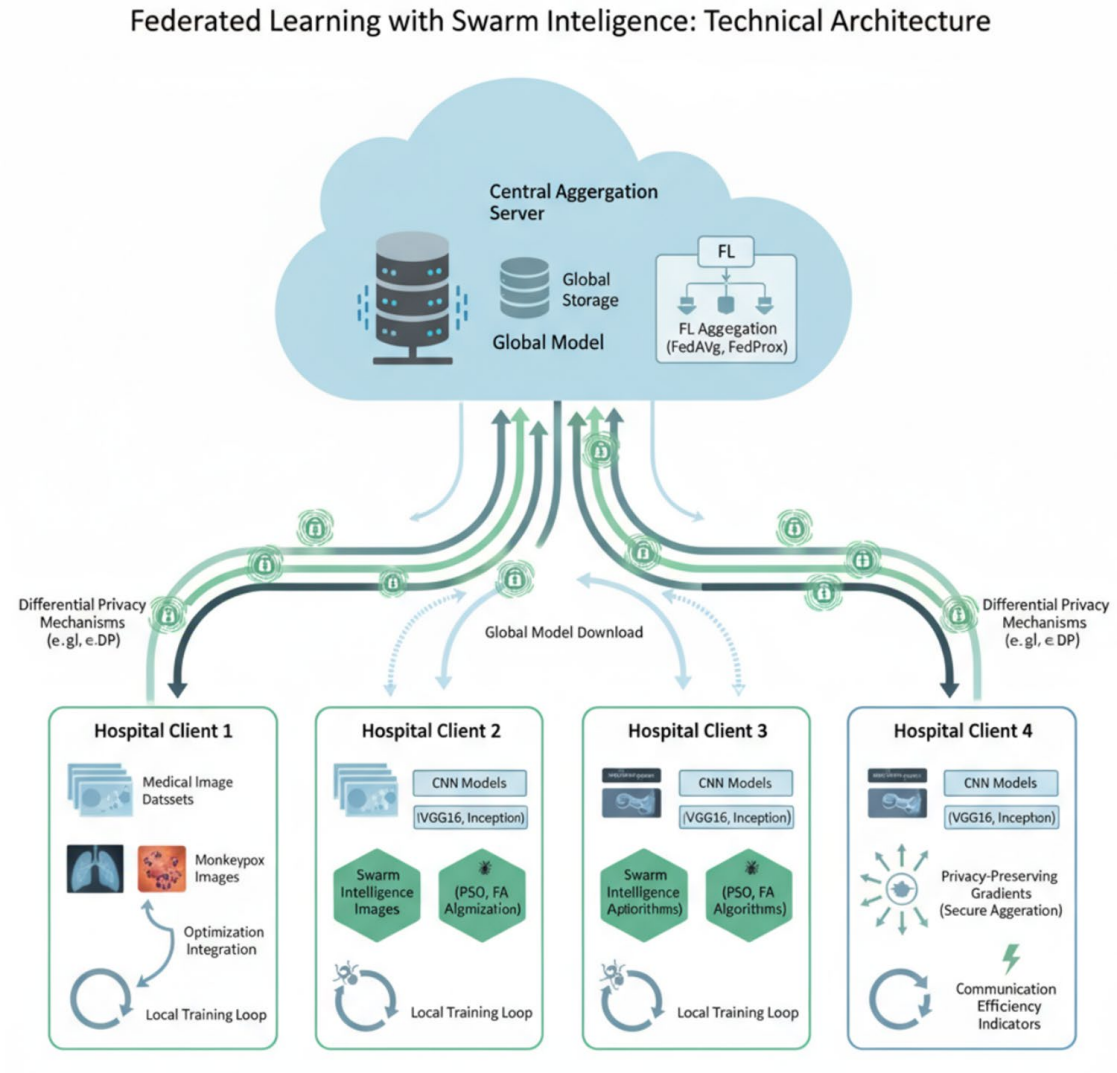
Federated Learning Framework Architecture showing the communication flow between the central server and distributed clients. The framework ensures privacy preservation through local model training and parameter-only sharing.

The proposed federated learning system operates through a structured client-server architecture where multiple healthcare institutions collaborate without sharing sensitive patient data. Figure 1 illustrates the comprehensive information flow between the central server and distributed federated clients within the proposed system.

The system architecture demonstrates how local models at each client institution train on private medical data while only sharing model parameters with the central server. This approach maintains data locality while enabling collaborative learning across multiple healthcare providers. The central server performs model aggregation without accessing raw medical images, ensuring compliance with healthcare privacy regulations.

**Fig. 2:**
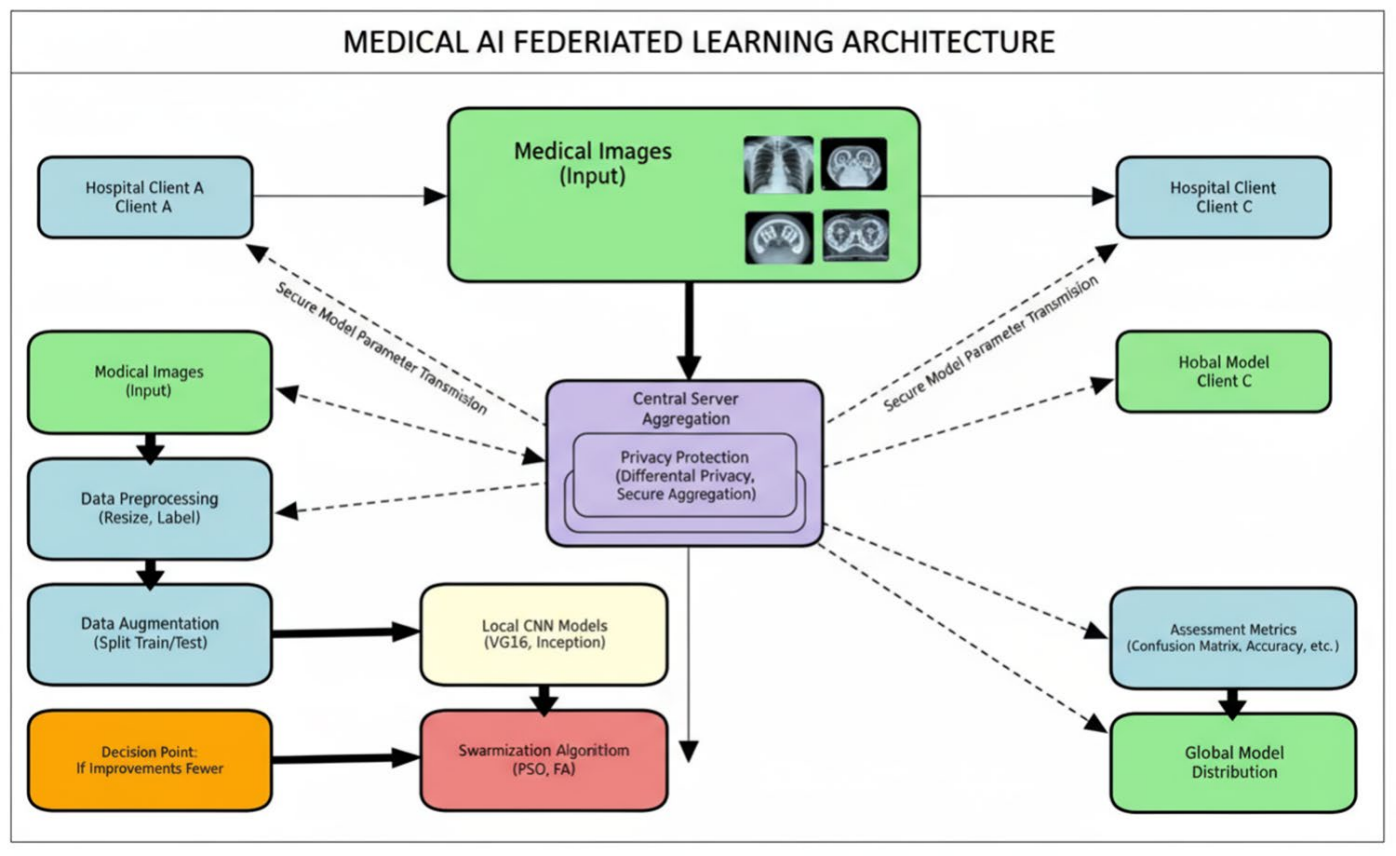
Methodology flowchart depicting the complete workflow from medical image input through data preprocessing, augmentation, ensemble CNN model training, and swarm intelligence optimization to final enhanced accuracy output.

Figure 2 presents the detailed methodology workflow, demonstrating the integration of data preprocessing, augmentation techniques, ensemble CNN model training, and swarm intelligence optimization for enhanced classification performance.

The primary objective is to demonstrate that deep CNN models can be utilized in an FL setup, allowing models to learn from diverse and private datasets without compromising data security. The study focuses on optimizing CNN architecture and algorithms to improve performance and prediction accuracy. The key contributions of this paper are as follows:

- *Privacy-Aware Federated Learning Framework* We have developed a framework designed to predict three types of medical conditions (COVID-19, breast cancer, and monkeypox) through FL, ensuring that sensitive medical data remains on local user devices while the global model is updated on a central server.
- *Optimization Algorithms for Feature Selection* At the client level, PSO and FA are employed to optimize feature selection. These algorithms help improve the predictive accuracy of the model and reduce classification errors.
- *Federated Matched Aggregation Algorithm* We propose a Federated Matched Aggregation-based algorithm, which ensures that the global model can effectively aggregate local models while preserving privacy. This allows the system to benefit from the diverse datasets across clients without the need to share raw data.
- *Evaluation and Validation* The proposed framework has been tested on real-world medical datasets. Performance is evaluated in terms of prediction accuracy, classification errors, and communication efficiency, with comparisons to state-of-the-art FL algorithms.

Each component of the framework, including data preprocessing, augmentation, CNN model training, and optimization, is detailed in the following sections. The workflow enables the aggregation of locally trained models into an accurate global model, ensuring enhanced performance and data privacy.

### Dataset description

The proposed model has been implemented on three distinct databases, encompassing a wide variety of medical images, as outlined in Table 2. Given the limited availability of data, the augmentation process is crucial for expanding the training and testing datasets. Image resizing plays an important role in reducing computational costs and standardizing image dimensions for subsequent processing layers. Data augmentation also helps mitigate overfitting by reorganizing and balancing the dataset, which is especially beneficial when the initial training set is small. This process improves model accuracy while reducing the costs associated with labeling and cleaning the original dataset.

**Table 2:** Database information and federated client distribution.

Dataset	Total	After Aug.	Client Distribution							
			Client A		Client B		Client C		Client D	
			N	A	N	A	N	A	N	A
COVID-19^39^	5,856	58,560	12,000	3,000	13,000	2,500	8,000	3,030	15,000	2,030
Monkeypox^40^	569	5,690	1,000	250	1,100	350	1,200	450	1,040	300
Breast cancer^41^	320	3,200	610	190	750	150	500	100	700	200

The dataset distribution strategy ensures realistic heterogeneity across federated clients, mimicking real-world scenarios where different healthcare institutions possess varying amounts of medical data. This heterogeneous distribution enables comprehensive evaluation of the proposed framework’s robustness across diverse data availability scenarios.

#### COVID-19 dataset

The chest lesions caused by COVID-19 have severely impacted human health due to the virus’s rapid spread. Researchers have identified these lesions as one of the most catastrophic health challenges, currently affecting millions globally ^23^. COVID-19 spreads through respiratory droplets when infected individuals sneeze, cough, or talk, making early detection of chest lesions essential for limiting the virus’s transmission. Given the widespread nature of COVID-19, there is a pressing need for quick and accurate detection methods. The World Health Organization (WHO) has declared COVID-19 chest lesions a public health emergency due to their severity and global impact ^42^. Currently, three methods are used to detect chest lesions caused by COVID-19:

- *Computed Tomography Scans* Produces 3D radiographic images.
- *Reverse Transcription Polymerase Chain Reaction (RT-PCR)* Detects viral RNA from nasal swabs.
- *CXR* Offers a more portable and efficient solution, taking only about 15 seconds per scan ^43^.

The COVID-19 dataset is divided into two folders, for training and testing, each containing subfolders for Pneumonia and Normal image categories. It includes 5,856 chest X-ray images in JPEG format, classified into these two groups ^39^. The images were taken from pediatric patients aged one to five at the Guangzhou Women and Children’s Medical Center. All images underwent quality control before analysis, being evaluated by two expert physicians, with a third expert review to mitigate grading errors.

While this dataset was collected prior to the COVID-19 pandemic and originally intended for pneumonia detection, it has been widely adopted in federated learning studies for COVID-19-related tasks^20,21,23^. This is because viral pneumonia patterns in chest X-rays exhibit radiographic similarities to COVID-19 manifestations, making the differentiation from healthy tissue a clinically relevant proxy. Moreover, using a pre-pandemic dataset avoids privacy concerns associated with patient data collected during the public health emergency.

**Fig. 3:**
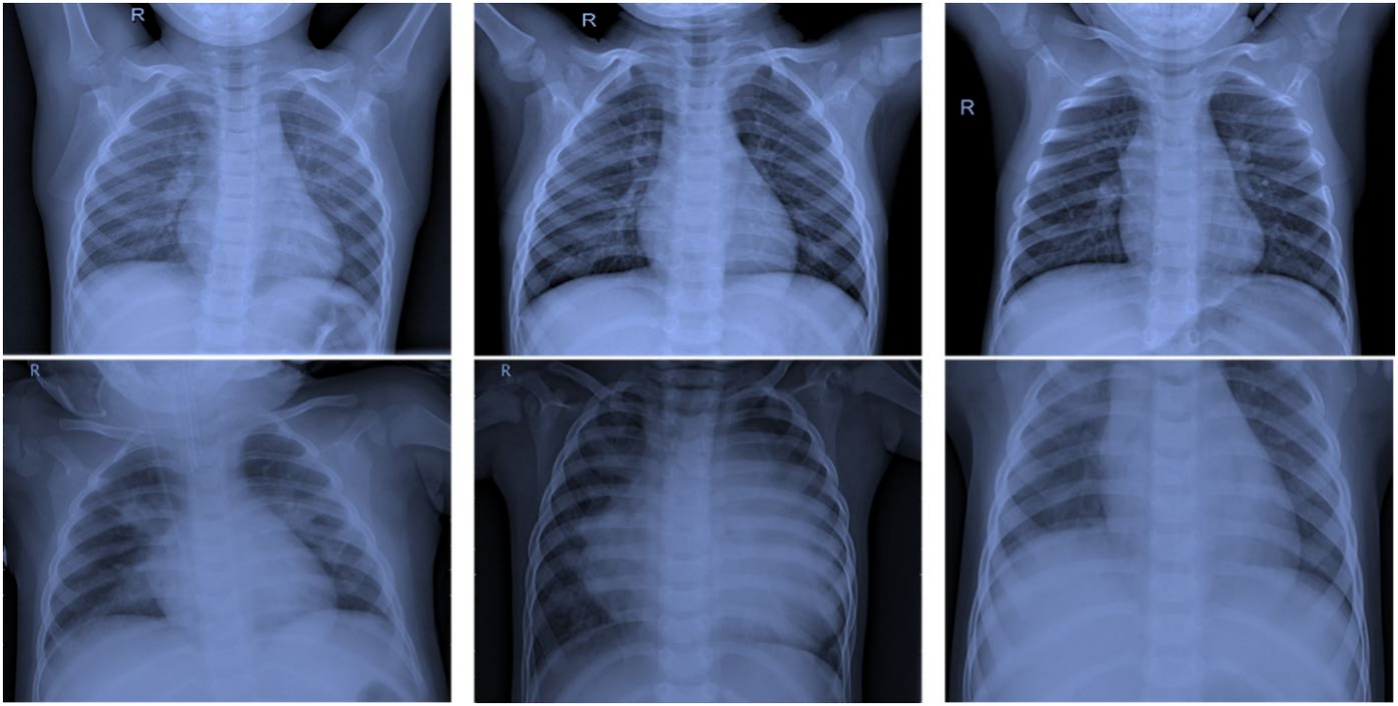
Samples of chest X-rays in patients. The first row is for normal patients, and the second row is for infected individuals.

Figure 3 presents examples of chest X-rays from COVID-19 patients. These images show clear lungs without abnormal opacification, while Pneumonia manifests as focal lobar consolidation or a diffuse “interstitial” pattern in both lungs.

#### Monkeypox dataset

In addition to COVID-19, the spread of other zoonotic diseases, such as monkeypox, presents a serious threat to global health. Monkeypox (MPX) is a zoonotic virus first discovered in 1958 during vaccine research in Denmark, and it was named after the first infected monkey ^44^. In 2022, monkeypox was classified as a global outbreak. The virus can be transmitted to humans through direct contact with infected animals, and common symptoms include fever, muscle aches, headache, swollen lymph nodes, and rash.

Polymerase Chain Reaction (PCR) tests are typically used for accurate diagnosis, while antiviral medications like tecovirimat, cidofovir, and brincidofovir are used to manage symptoms. Although there is no specific vaccine for monkeypox, smallpox vaccines have been used to bolster immunity. Researchers are also using artificial intelligence to aid in the diagnosis and classification of monkeypox, assisting healthcare professionals. Nearly 70 countries have been affected by this outbreak, impacting communities with diverse backgrounds and age groups ^45^.

The monkeypox dataset^40^ contains 569 skin lesion images and was the first publicly available dataset for this emerging zoonotic disease, released on Kaggle in 2022. In this study we employ this initial version to maintain consistency with prior federated learning investigations that used the same source^24–27^, enabling direct comparability of results. We note that an expanded and verified version (MSLD-v2) has since been released; validating our framework on this newer dataset is a direction for future work, as discussed in Sect. 5.4.

**Fig. 4:**
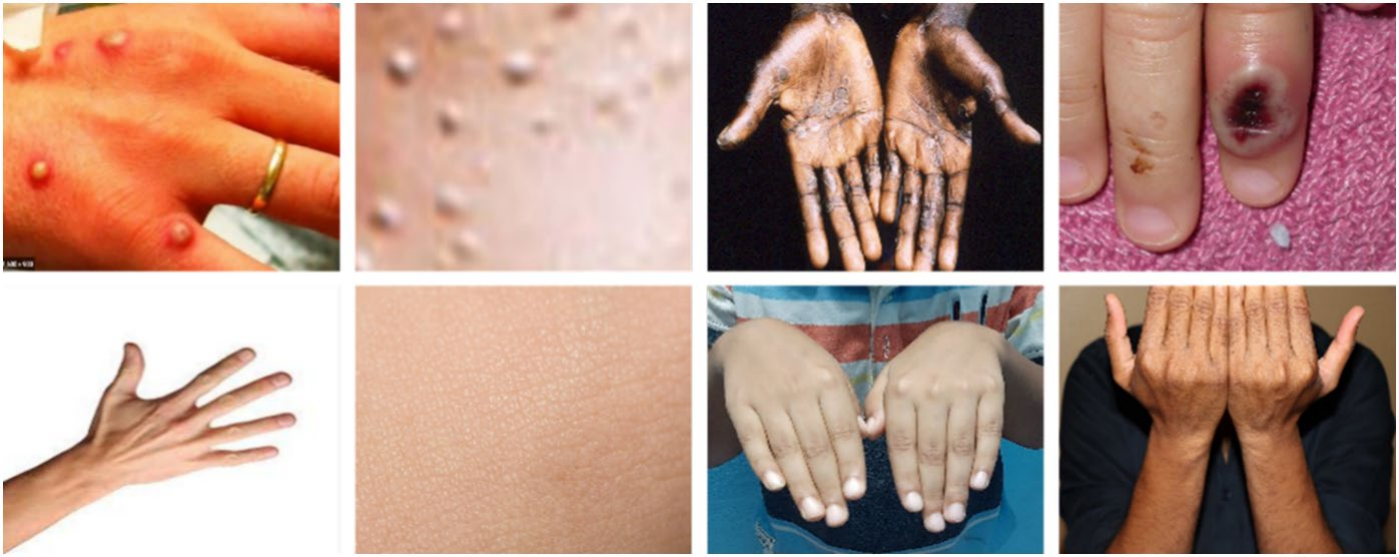
Samples of patients with monkeypox. The first row displays samples from abnormal patients, while the second row shows samples from normal individuals.

Figure 4 presents examples of monkeypox in patients, showing the characteristic skin lesions and normal skin samples for comparison.

#### Breast cancer dataset

Breast cancer originates in the tissue of the breast, typically in the lining of the milk ducts or lobules and can spread throughout the body. It is the second most common type of cancer globally, making the early and accurate detection of breast cancer crucial for improving treatment outcomes and survival rates. Recent analyses show a marked increase in breast cancer cases over the past decade. Medical imaging techniques play a vital role in diagnosing and treating breast cancer, as they allow healthcare providers to detect abnormalities and identify the presence of malignant cells.

Early detection is particularly important because cancerous cells can spread through the bloodstream or lymphatic system, posing further risks during biopsy or surgery. Specialized imaging techniques can help focus on the breast profile by removing background noise and pectoral muscle tissue, thereby enhancing detection.

Performing regular self-exams is important for identifying any changes in the breast, and any irregularities should be promptly evaluated by a healthcare professional. The first sign of breast cancer in some individuals may be a new lump or mass in the breast, which may be painless, hard, and have uneven edges—features more likely to be cancerous. However, in some cases, cancerous lumps can also be tender, soft, or rounded. Symptoms of breast cancer vary widely, and some cases may not present with obvious signs. According to the American Cancer Society, potential symptoms of breast cancer include unusual swelling, skin irritation, breast or nipple pain, nipple discharge, or a lump in the underarm area ^46^.

The breast cancer dataset^41^ comprises 320 mammogram images from the Wisconsin Diagnostic Breast Cancer collection, a well-established benchmark in medical machine learning research. Despite its modest size, this dataset offers a challenging testbed for federated learning due to the subtle radiographic features distinguishing malignant from benign tissue.

**Fig. 5:**
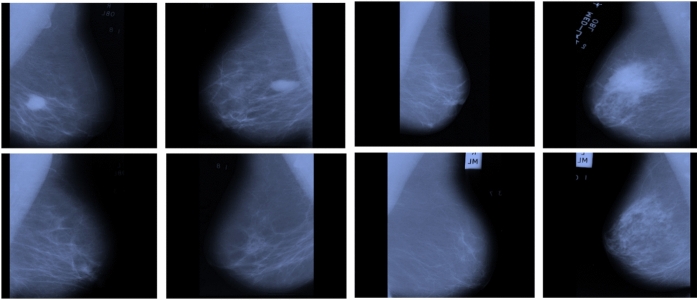
Displays samples of breast cancer patients. The first row shows samples from patients with abnormal conditions, while the second row displays samples from normal individuals.

Figure 5 presents examples of mammograms showing breast cancer in patients, demonstrating the radiological differences between malignant and normal breast tissue.

### Deep CNN model

The core concepts in a Convolutional Neural Network (CNN) are as follows:

- *Input layer* This layer processes the input image by taking in a set of pixels that belong to the image data.
- *Convolutional layer* The convolution process extracts features by applying filters to the input image, creating a feature map. This reduces the storage space required for each image. The convolutional layer takes the output of the previous layer as input and performs convolution operations. It is the core component of CNNs, playing a key role in reducing the network’s complexity. Three important hyperparameters govern the convolutional layer: depth, stride, and zero-padding.
- *Pooling layer* Executed after each convolutional layer, the pooling layer summarizes the features in the feature map using a 2D filter. This reduces the dimensionality of the feature map, decreasing the number of parameters to be learned and the operations performed. Two common types of pooling are max pooling and average pooling.
- *Fully connected layer* Often referred to as an assembler, this layer connects the features from all preceding layers to perform classification. It is the final layer, aggregating data from earlier layers and feeding it into the output layer, although it is computationally intensive.

CNN models used in the proposed method:

- *VGG16* This network utilizes multiple small $3 \times 3$ convolution kernels instead of a single large kernel, which is preferable for achieving a broader receptive field. Using smaller kernels increases network depth, thereby enabling the learning of more complex patterns while reducing computational costs ^47^.
- *Inception* This model, which won the 2014 ILSVRC, contains multiple convolution kernels of different sizes ($1 \times 1$, $3 \times 3$, and $5 \times 5$) within each inception module. The interleaved $1 \times 1$ convolutional layers reduce the dimensionality of the feature space. The model is composed of nine interconnected inception modules ^48^.

### Feature selection

Swarm Intelligence (SI), inspired by the collective behaviors observed in nature, has gained significant attention in Artificial Intelligence (AI). Social swarms such as ants, bees, and schools of fish display coordinated self-organizing behavior despite consisting of relatively simple individuals. For example, ants find the shortest path between food sources and their nest through chemical pheromone trails, while bees use a “waggle dance” to communicate the location of new food sources ^14^.

#### Firefly algorithm

Developed by Yang in 2008 ^10^, the Firefly Algorithm (FA) is a nature-inspired, stochastic, meta-heuristic approach used for solving complex optimization problems. It is based on the behavior of fireflies’ flashing lights and finds solutions through trial and error. FA has been widely applied in optimization across various domains. To adapt the algorithm for specific problems, modifications or hybridizations are often employed.

In FA, two key factors are the range of light intensity and the concept of attractiveness. The light intensity ($I$) at a given location represents the solution’s fitness, and it decays with distance according to Eq. (1):

1 $$\begin{aligned} I = I_0 e^{-\gamma r} \end{aligned}$$

where $I_0$ is the light intensity at the source, and $\gamma$ is the light absorption coefficient. Attractiveness ($\beta$) is directly proportional to the light intensity and follows:

2 $$\begin{aligned} \beta = \beta _0 e^{-\gamma r^2} \end{aligned}$$

The Euclidean distance between two fireflies $i$ and $j$ is calculated as shown in Eq. (3):

3 $$\begin{aligned} r_{ij} = \sqrt{\sum _{k=1}^{d} (x_{i,k} - x_{j,k})^2} \end{aligned}$$

The movement of firefly $i$ toward a more attractive firefly $j$ is modeled in Eq. (4):

4 $$\begin{aligned} x_i = x_i + \beta _0 e^{-\gamma r_{ij}^2} (x_j - x_i) + \alpha (\text {rand} - 0.5) \end{aligned}$$

When $\alpha$ is a random number drawn from a Gaussian distribution, the movement of a firefly consists of three terms: the current position of the firefly, attraction to another more attractive prey, and a random walk that includes a randomization parameter and a randomly generated number from the interval $[0,1]$. When $\beta = 0$, the movement depends on the random walk only. On the other hand, the parameter $\alpha$ has a crucial impact on the convergence speed. The objective function to be optimized can be associated with these phenomena. As a result, the base FA can be formulated as illustrated in Algorithm 1.

**Algorithm 1:**
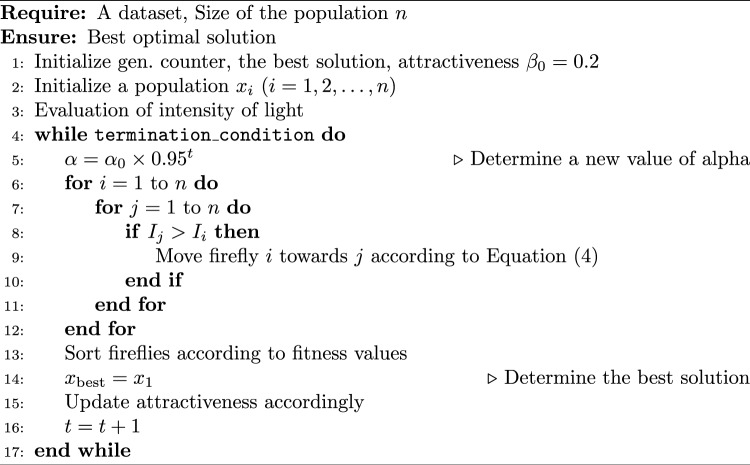
Base Firefly algorithm

The firefly population is initialized using the random initialization function, typically performed randomly. The firefly search process involves the while loop (lines 4-12 in Algorithm 1) and includes the following steps:

1. The alpha modification function modifies the initial parameter value (optional in the FA algorithm).
2. The fitness evaluation function assesses solution quality, implementing the fitness function $f(s)$.
3. The sorting function sorts the FA population based on fitness values.
4. The best selection function selects the best individual in the population.
5. The movement function moves FA positions in the search space, moving them towards more attractive individuals.

#### PSO algorithm

The Particle Swarm Optimization (PSO) algorithm, developed in 1995 by Kennedy and Eberhart ^8^, is inspired by the behavior of animal groups like bird and fish swarms. PSO is an iterative global optimization method known for its simplicity, scalability, robustness, and fast convergence. The algorithm uses particles (representing potential solutions) to search for the optimal solution, updating each particle’s position and velocity based on individual and collective experiences.

Each particle updates its velocity according to Eq. (5):

5 $$\begin{aligned} v_{i}^{t+1} = w \cdot v_{i}^{t} + c_1 \cdot r_1 \cdot (p_{i}^{t} - x_{i}^{t}) + c_2 \cdot r_2 \cdot (g^{t} - x_{i}^{t}) \end{aligned}$$

where $w$ is the inertia weight, $c_1$ and $c_2$ are constants, and $r_1$ and $r_2$ are random numbers. The new position is updated as per Eq. (6):

6 $$\begin{aligned} x_{i}^{t+1} = x_{i}^{t} + v_{i}^{t+1} \end{aligned}$$

where $v_{i}^{t}$ is the velocity of the particle or agent, $p_{i}^{t}$ is Personal Best, and $g^{t}$ is Global Best.

Equation (5) defines the variable $w$ as inertia, indicating that a particle moves in the same direction and with the same velocity. The parameter $w$ represents the inertia weight, a positive constant that is crucial for balancing global search (exploration) and local search (exploitation). The variable $c_1$ represents personal influence, indicating an improvement in the individual by making the particles return to a previous position that is better than the current one. The variable $c_2$ represents social influence, indicating that the particle follows the best neighbor’s direction. The inertia variable involves diversification, which means searching for new solutions and finding the region with potentially the best solutions. Both social and personal influences contribute to intensification, involving the exploration of previous solutions and the identification of the best solution for a specific region.

**Algorithm 2:**
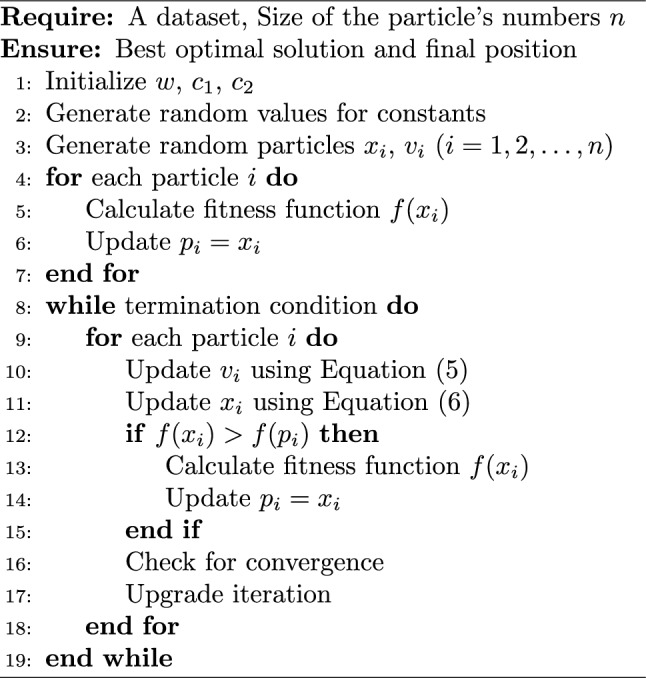
Base PSO algorithm

In Algorithm 2, the particles start their search for the best solution by randomly selecting a location within the solution space. They then embark on a journey toward the optimal solution, drawing from both their individual experiences and the collective knowledge of the swarm.

According to the algorithm steps for particle swarm optimization, there are three important points to consider: parameters ($w$, $c_1$, $c_2$), particle number, and fitness function. The proper definition of the fitness function is crucial for the use of meta-heuristic algorithms, as problems are solved by converting them into fitness functions that indicate the distance of the particles to the best solution. $v_i^t$ represents the current velocity of the particle and $x_i^t$ denotes the position. The convergence of the particle depends on these two parameters. Therefore, the convergence of the particle to the solution is highly dependent on these parameters, making them extremely important. The number of particles directly impacts the solution, as an increase in the number of particles improves the solution but slows down the operation of the algorithm.

### Feature selection and optimization rationale

The feature selection process in this study employs Particle Swarm Optimization (PSO) and Firefly Algorithm (FA) due to their proven advantages in handling complex optimization problems. While several optimization techniques, such as Genetic Algorithms (GA), are available, the choice of PSO and FA is motivated by the following factors:

#### Simplicity and efficiency

PSO and FA are computationally less intensive compared to GA, making them more suitable for large-scale problems like federated learning, where multiple local models are optimized simultaneously. The simpler implementation of PSO and FA reduces the risk of excessive computational overhead, particularly in scenarios involving iterative feature selection processes.

#### Convergence speed

PSO is known for its rapid convergence to optimal or near-optimal solutions, which is essential for reducing the time required to optimize local models in a federated learning framework. FA exhibits strong exploratory capabilities, quickly identifying relevant features while avoiding local minima, thus ensuring robust model performance.

#### Scalability and adaptability

Both PSO and FA are easily adaptable to multi-objective optimization tasks, aligning well with the goals of federated learning, where trade-offs between accuracy, communication cost, and privacy must be managed. The adaptability of PSO and FA to high-dimensional data makes them particularly effective for medical image analysis, where datasets often involve thousands of features.

#### Comparative advantages over GA

GA, while powerful, requires more complex encoding and decoding of solutions, leading to higher computational demands. GA’s crossover and mutation operations introduce additional randomness, which can prolong the optimization process compared to the guided search mechanisms of PSO and FA. PSO and FA offer a more intuitive parameter-tuning process, making them easier to integrate and fine-tune in the federated learning context.

#### Success in related domains

Both PSO and FA have demonstrated superior performance in various medical image analysis tasks, such as disease detection and classification, providing a strong empirical basis for their selection. Prior research highlights their effectiveness in identifying optimal feature subsets, which directly contributes to improved classification accuracy and reduced model complexity.

By leveraging the strengths of PSO and FA, this study ensures an efficient and effective feature selection process tailored to the unique challenges of federated learning in medical image analysis.

### Federated learning

Federated learning emerged from Google’s pioneering research in 2016, addressing the fundamental challenge of training machine learning models across distributed data sources without centralizing sensitive information ^2^. This paradigm has gained significant traction in academic and industrial research, offering practical solutions for collaborative machine learning across institutional boundaries. Applications span diverse domains including Internet of Things ecosystems, industrial automation, healthcare systems, and distributed computing environments ^49^.

#### Federated learning layered architecture

Modern federated learning implementations adopt a structured architectural approach comprised of four interconnected layers, each serving specialized functions within the collaborative framework:

- *Application Layer* Individual healthcare institutions manage local data processing workflows, conduct CNN model training using institutional datasets, and execute specialized optimization procedures tailored to medical imaging requirements.
- *Federation Layer* Centralized coordination mechanisms oversee global model synthesis, manage parameter synchronization across participating institutions, and conduct comprehensive performance assessments throughout the learning process.
- *Communication Layer* Sophisticated encryption protocols facilitate secure parameter transmission between participating nodes while implementing differential privacy safeguards to protect sensitive medical information during inter-institutional exchanges.
- *Infrastructure Layer* Distributed computational resources spanning healthcare networks provide the foundational support for federated operations, enabling scalable deployment across diverse institutional environments.

This architectural framework ensures functional separation while maintaining cohesive integration across all operational levels. The application layer handles sensitive medical data processing within institutional boundaries, while the federation layer orchestrates distributed learning coordination. The communication layer establishes secure channels for parameter exchange, and the infrastructure layer provides robust computational foundations. Such layered organization facilitates privacy-preserving collaborative learning among healthcare institutions with varying technological capabilities and regulatory requirements.

Healthcare environments present unique challenges for machine learning deployment, particularly regarding data utilization restrictions imposed by confidentiality requirements and regulatory compliance. These constraints often prevent healthcare institutions from fully leveraging their accumulated data assets. Federated learning addresses these limitations by enabling collaborative model development without requiring direct data sharing, thus maintaining institutional data sovereignty while fostering knowledge exchange.

The federated learning workflow follows established patterns across multiple iterations. Initially, a foundational model resides on a central coordination server, with identical copies distributed to participating client institutions. Each institution conducts local training using its proprietary datasets, resulting in specialized model adaptations reflecting local population characteristics and clinical practices.

Following local training phases, participating institutions share model parameter updates through secure aggregation mechanisms. The central coordination server synthesizes these contributions using weighted averaging techniques, generating enhanced global models that incorporate knowledge from all participating sites. This collaborative approach improves model generalizability across diverse patient populations while respecting institutional privacy boundaries.

The updated global model undergoes redistribution to participating clients for subsequent training iterations. Each cycle progressively refines the collaborative model while maintaining strict data privacy protocols, enabling continuous improvement without compromising institutional confidentiality requirements.

FL architectures differ fundamentally from conventional centralized approaches through their emphasis on distributed data processing. Rather than aggregating datasets at central locations, federated systems coordinate model training across multiple sites while preserving data locality. Central servers manage coordination functions, addressing critical challenges including data privacy preservation, security maintenance, access control implementation, and heterogeneity management. Each participating institution maintains local copies of global models, represented through model weight parameters ($\omega$), conducting training using local datasets before transmitting parameter updates for global aggregation. This approach eliminates the necessity for raw data sharing while enabling collaborative knowledge development.

The mathematical foundation of federated learning centers on distributed optimization objectives, formulated as:

7 $$\begin{aligned} \min _{\omega } F(\omega ) = \sum _{k=1}^{K} \frac{n_k}{n} F_k(\omega ) \end{aligned}$$

This formulation seeks to minimize the aggregate loss function across $k$ participating clients through systematic optimization of model parameters $\omega$, where individual client contributions are weighted according to their data contributions.

The Federated Averaging algorithm, developed by McMahan and colleagues, represents a foundational approach to distributed model aggregation. Under this framework, each participating client receives current global model parameters $\omega _t$ from the coordination server. Clients subsequently compute gradient averages using local datasets and update model weights through stochastic gradient descent procedures. The coordination server aggregates updated parameters using weighted averaging:

8 $$\begin{aligned} \omega _{t+1} = \sum _{k=1}^{K} \frac{n_k}{n} \omega _k^{t+1} \end{aligned}$$

Here, $n_k$ denotes the quantity of data samples utilized by client $k$ during local training. Communication overhead reduction strategies often involve multiple local gradient descent iterations before parameter transmission and global aggregation, though such approaches may influence FedAvg convergence characteristics.

**Algorithm 3:**
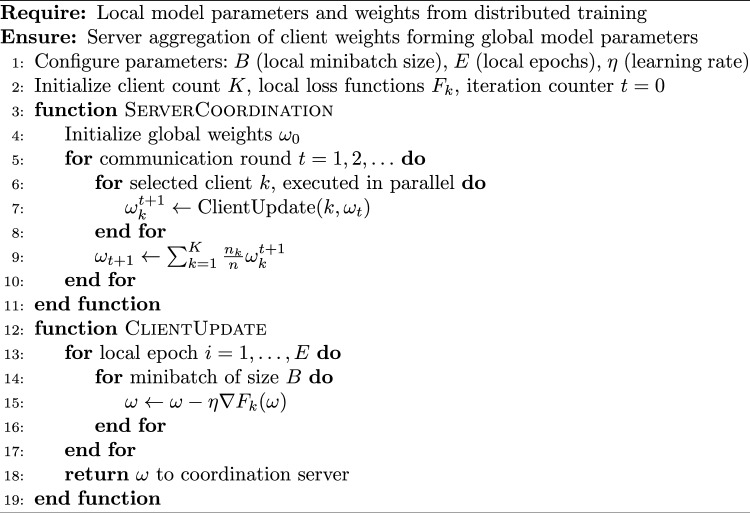
Federated Averaging Algorithm Implementation

Algorithm 3 outlines the systematic process of federated model training, assuming appropriate dataset normalization procedures. Client institutions execute local parameter updates, transmitting results to the coordination server for global aggregation. This iterative process continues across predetermined communication rounds until convergence criteria are satisfied.

Healthcare data management faces substantial challenges due to stringent privacy requirements and restricted access protocols, creating significant barriers for large-scale machine learning deployment across global healthcare networks. Federated learning provides viable solutions by enabling secure model training using patient data from multiple institutions while maintaining strict data locality requirements. This approach promotes inter-institutional collaboration while allowing models to incorporate diverse demographic and clinical patterns without compromising patient privacy or regulatory compliance.

Furthermore, federated learning methodologies can provide valuable insights into patient populations and disease patterns across diverse geographical regions, enabling smaller healthcare facilities and rural hospitals to access advanced diagnostic technologies. This democratization of healthcare AI has demonstrated practical success in various applications, including clinical outcome prediction for COVID-19 patients across international healthcare networks ^50^.

### Swarm intelligence integration within federated learning frameworks

The incorporation of swarm intelligence methodologies, particularly PSO and FA, within FL architectures represents an innovative approach for addressing inherent challenges in distributed medical image analysis while maintaining robust privacy preservation. This integration leverages bio-inspired optimization principles to enhance model performance characteristics, reduce communication overhead, and accelerate convergence across distributed healthcare environments.

#### Multi-level optimization framework

Our proposed integration methodology operates through a comprehensive multi-level optimization paradigm where swarm intelligence algorithms simultaneously address multiple aspects of the FL process. Unlike conventional FL implementations that employ static hyperparameter configurations, this approach incorporates dynamic parameter adaptation based on local data characteristics and global performance feedback mechanisms.

The swarm intelligence integration manifests across three complementary operational levels:

- *Local Client Optimization* Individual participating healthcare institutions employ PSO or FA algorithms to optimize local model hyperparameters, including learning rate schedules, batch size configurations, dropout rate adjustments, and architectural parameter selections tailored specifically for medical imaging classification tasks.
- *Feature Selection Optimization* Swarm intelligence algorithms systematically identify discriminative features from high-dimensional medical image representations, effectively reducing communication overhead between clients and servers while preserving diagnostic accuracy across diverse pathological conditions.
- *Global Aggregation Optimization* The central coordination server employs swarm intelligence methodologies to optimize aggregation weight distributions, ensuring appropriate weighting of client contributions based on local data quality assessments and individual model performance characteristics.

#### Multi-objective optimization formulation

The fitness function serves as the foundational guiding principle for swarm intelligence optimization, incorporating multiple objectives essential for effective federated medical image analysis. Our comprehensive fitness function formulation addresses the complex trade-offs inherent in distributed healthcare AI systems:

9 $$\begin{aligned} \begin{aligned} \text {Fitness}(\theta ) = \alpha _1 \cdot \text {Accuracy}(\theta ) - \alpha _2 \cdot \text {CommCost}(\theta ) - \alpha _3 \cdot \text {ConvergenceTime}(\theta )\\\\ + \alpha _4 \cdot \text {Privacy}(\theta ) - \alpha _5 \cdot \text {ModelComplexity}(\theta ) \end{aligned} \end{aligned}$$

where $\theta$ represents the comprehensive parameter vector encompassing hyperparameters, feature selection variables, and architectural configuration choices. The coefficients $\alpha _1, \alpha _2, \alpha _3, \alpha _4, \alpha _5$ undergo dynamic adjustment to balance competing objectives throughout the optimization process.

The accuracy component utilizes F1-score metrics to address class imbalance issues commonly encountered in medical datasets:

10 $$\begin{aligned} \text {Accuracy}(\theta ) = \frac{2 \cdot \text {Precision}(\theta ) \cdot \text {Recall}(\theta )}{\text {Precision}(\theta ) + \text {Recall}(\theta )} \end{aligned}$$

Communication cost assessment incorporates model size, feature dimensionality, and update frequency considerations:

11 $$\begin{aligned} \begin{aligned} \text {CommCost}(\theta ) = \beta _1 \cdot \text {ModelSize}(\theta ) + \beta _2 \cdot \text {FeatureDim}(\theta ) \\ \quad + \beta _3 \cdot \text {UpdateFreq}(\theta ) \end{aligned} \end{aligned}$$

**Algorithm 4:**
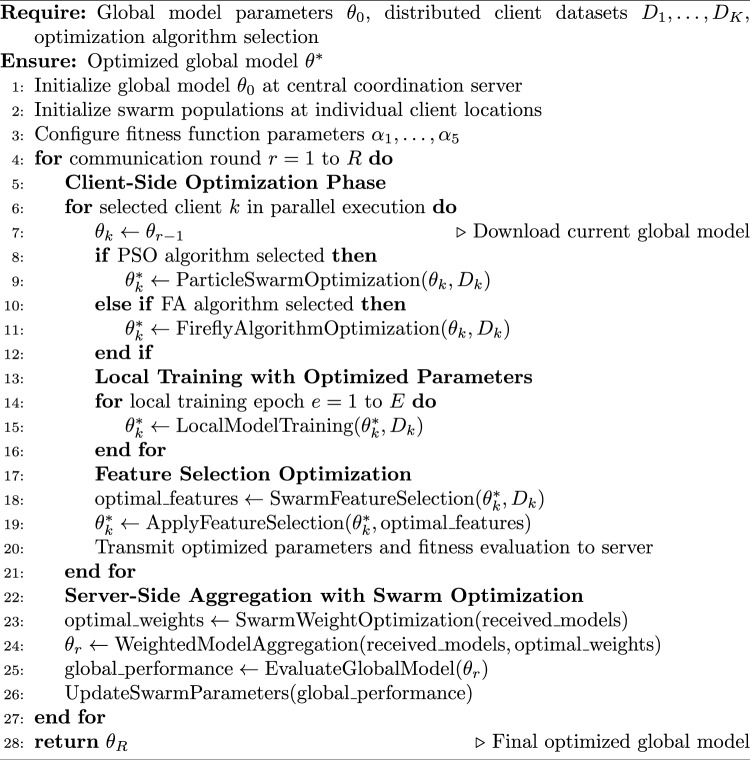
Enhanced Federated Learning with Swarm Intelligence Integration

This comprehensive methodology combines swarm intelligence optimization with federated learning principles to address the distinctive challenges of distributed medical image analysis. Through the strategic integration of PSO and FA algorithms with CNN architectures within privacy-preserving federated frameworks, this approach enables collaborative healthcare AI development while maintaining patient data confidentiality and regulatory compliance standards. The experimental validation framework ensures thorough evaluation of proposed techniques across diverse medical imaging scenarios, establishing a robust foundation for practical deployment in real-world healthcare environments.

### Security considerations and attack mitigation

Federated learning architectures operating across distributed healthcare networks inherently present complex security challenges that require careful consideration of both technological vulnerabilities and regulatory compliance frameworks. The multi-layered nature of these systems creates diverse attack surfaces, each demanding specialized defensive strategies tailored to the sensitive nature of medical data and the critical importance of diagnostic accuracy.

The healthcare domain imposes unique constraints on security implementation, where patient privacy protection must be balanced against the need for collaborative learning and emergency access requirements. These considerations become particularly crucial when multiple healthcare institutions participate in federated training while maintaining compliance with varying international privacy regulations and medical device standards.

**Table 3:** Security Attacks and Mitigation Solutions in Federated Learning Layers.

Attack Vector	Layer	Clinical Impact	Mitigation Solution
*Application Layer*			
Model Poisoning	Application	Diagnostic misclassification	Byzantine-resilient aggregation
Data Poisoning	Application	Systematic diagnostic bias	Multi-institutional validation
Gradient Leakage	Application	Patient privacy breach	Differential privacy ($\varepsilon = 1.9$)
*Federation Layer*			
Byzantine Faults	Federation	Global model corruption	Reputation-based weighting
Free-riding	Federation	Degraded performance	Contribution assessment
Model Inversion	Federation	Patient data exposure	Parameter obfuscation
*Communication Layer*			
Eavesdropping	Communication	Model update exposure	End-to-end encryption (AES-256)
Man-in-the-Middle	Communication	Corrupted parameters	Digital signature verification
Replay Attacks	Communication	Outdated model states	Timestamp validation
*Infrastructure Layer*			
DDoS Attacks	Infrastructure	System unavailability	Traffic pattern analysis
Sybil Attacks	Infrastructure	Biased model development	Identity verification
Resource Depletion	Infrastructure	Service interruption	Resource monitoring
*Cross-Layer Attacks*			
Membership Inference	Multiple	HIPAA violations	k-anonymity preservation
Model Extraction	Multiple	IP theft	Access control enforcement

Our analysis identifies distinct vulnerability patterns across the four architectural layers, each presenting specific threat vectors that adversaries might exploit to compromise system functionality, patient confidentiality, or clinical decision-making processes. Table 3 provides a comprehensive mapping of identified threats, their potential healthcare implications, and the defensive measures incorporated into our framework design.

#### Rényi differential privacy accountant

To provide a formal privacy guarantee, we employ a Rényi Differential Privacy (RDP) accountant^51^ that tracks the cumulative privacy loss across multiple orders $\alpha$. Unlike the simpler moments accountant, RDP offers tighter bounds by considering a range of $\alpha$ values. We use $\alpha \in \{2,4,8,16,32,64,128\}$, which covers the typical spectrum for composition.

For each communication round, with sampling rate $q = 1/K$ (full client participation), the RDP of the subsampled Gaussian mechanism is bounded by:

$$\begin{aligned} D_\alpha (q,\sigma ) \le \frac{q^2 \alpha }{2\sigma ^2}, \quad \alpha \ge 2, \end{aligned}$$

where $\sigma$ is the noise multiplier. This bound is conservative and ensures that the actual privacy loss is no larger than the computed value.

The accountant maintains a cumulative RDP value for each $\alpha$. After *T* rounds, the total RDP is $T \cdot D_\alpha (q,\sigma )$. The final $(\varepsilon ,\delta )$-DP guarantee is obtained by minimizing over $\alpha$:

$$\begin{aligned} \varepsilon = \min _{\alpha } \left( T \cdot D_\alpha (q,\sigma ) + \frac{\log (1/\delta )}{\alpha -1} \right) . \end{aligned}$$

Algorithm 5 summarises the procedure for computing the noise multiplier $\sigma$ given a target $\varepsilon$ and for tracking the cumulative privacy loss during training.

**Algorithm 5:**
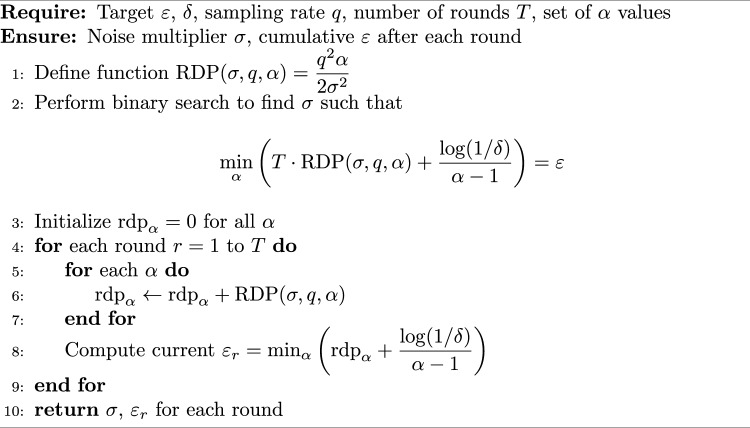
RDP Accountant for Federated Learning

For our experiments with target $\varepsilon = 1.9$, $\delta = 10^{-5}$, $q = 1/4$, and $T = 75$, the binary search yielded $\sigma = 1.12$. The cumulative $\varepsilon$ is plotted in Fig. 12.

## Experimental results and analysis

### Experimental configuration

The experimental validation was conducted on a high-performance computing platform equipped with an Intel(R) Core(TM) i7-1135G7 CPU @ 2.40GHz, supported by dual NVIDIA GeForce RTX 2070 Super GPUs (16 GB each) and 64 GB RAM. The implementation utilized TensorFlow and Keras frameworks to facilitate comprehensive deep learning-based medical image analysis. The primary objective focused on enhancing communication efficiency in federated learning systems while maintaining superior classification performance across diverse medical imaging tasks.

### Performance evaluation framework

To ensure statistical robustness, every experiment was repeated five times with distinct random seeds (42, 43, 44, 45, and 46). All results reported throughout this section are therefore presented as mean ± standard deviation over these five independent runs. This practice follows the reproducibility guidelines advocated in modern machine learning research and provides a realistic assessment of the variability inherent in stochastic processes such as mini-batch sampling, client participation, and the probabilistic updates of swarm intelligence algorithms.

The assessment framework employed confusion matrix analysis as the foundation for comprehensive performance evaluation. From the confusion matrix structure, For each run we derived critical performance metrics including Accuracy (ACC), Precision (PPV), Recall (Sensitivity), Specificity (SPE), F1-Score, and, where applicable, the area under the ROC curve (AUC). These metrics are derived from the confusion matrix using the standard formulas given in Equations (12)–(16). The primary metric for comparison is accuracy, but we also report F1-Score to account for class imbalance in the medical datasets.

12 $$\begin{aligned} & \text {Recall} = \frac{TP}{TP + FN} \times 100 \end{aligned}$$

13 $$\begin{aligned} & \text {Accuracy} = \frac{TP + TN}{TP + TN + FN + FP} \times 100 \end{aligned}$$

14 $$\begin{aligned} & \text {Specificity} = \frac{TN}{TN + FP} \times 100 \end{aligned}$$

15 $$\begin{aligned} & \text {F-Score} = \frac{2TP}{2TP + FP + FN} \times 100 \end{aligned}$$

16 $$\begin{aligned} & \text {Precision} = \frac{TP}{TP + FP} \times 100 \end{aligned}$$

where TP, TN, FP, and FN represent True Positive, True Negative, False Positive, and False Negative classifications, respectively.

### Hyperparameter configuration and optimization

The federated learning framework incorporated carefully selected hyperparameters optimized through systematic grid search methodology. Table 4 presents the comprehensive parameter configuration for both swarm intelligence algorithms and federated learning components.

**Table 4:** Hyperparameter configuration for experimental validation.

Algorithm	Parameter	Value
Firefly Algorithm	Iterations	75
	Population size	50
	Light absorption coefficient ($\gamma$)	1.0
	Initial attractiveness ($\beta _0$)	0.2
Particle Swarm Optimization	Iterations	75
	Population size	30
	Cognitive constant ($c_1$)	2.0
	Social constant ($c_2$)	2.0
	Inertia weight ($w$)	0.99 $\rightarrow$ 0.4
Federated Learning	Training epochs	100
	Batch size	60
	Network depth	15 layers
	Learning rate	0.001
	Client nodes	4
	Communication rounds	Variable

The hyperparameter selection process incorporated empirical validation through systematic experimentation. Iteration limits were established at 75 based on convergence analysis indicating diminishing returns beyond this threshold. Population sizes balanced exploration diversity with computational efficiency, employing 30 particles for PSO and 50 fireflies for FA. The inertia weight in PSO follows a linear decrease from 0.99 to 0.4 to facilitate convergence refinement.

### Client-specific performance analysis

#### Hospital model A performance evaluation

**Fig. 6:**
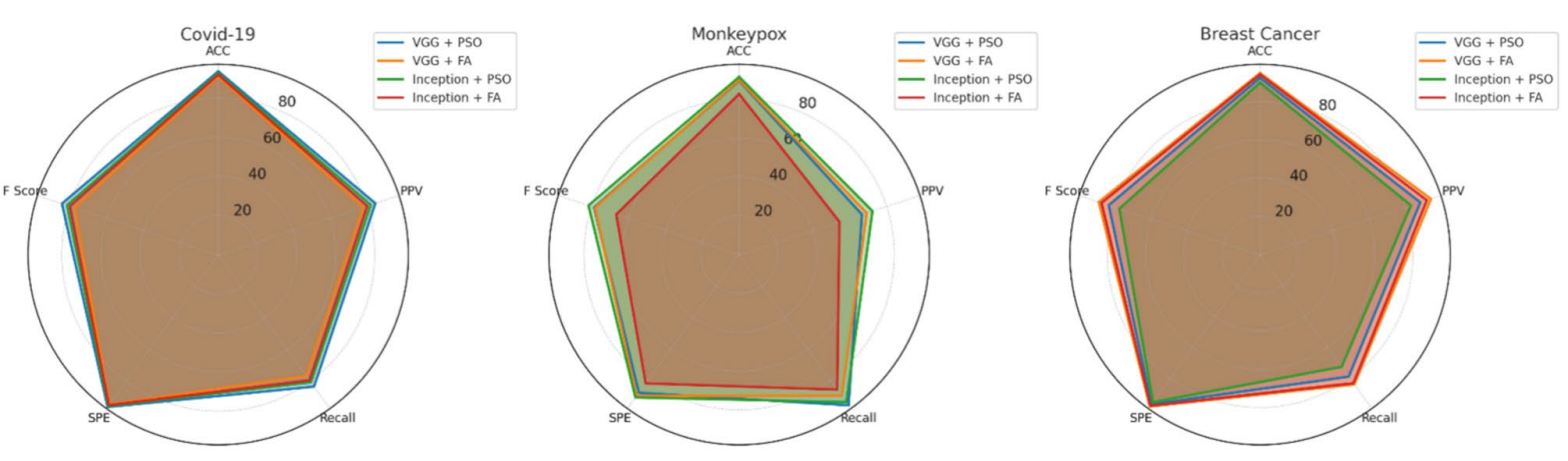
Performance analysis for Hospital Model A (Client A) across COVID-19, Monkeypox, and Breast Cancer datasets. The radar charts display accuracy, precision, recall, specificity, and F1-score metrics for different CNN-optimization combinations.

The experimental results for Client A demonstrate exceptional performance across all three medical datasets. Figure 6 presents the comprehensive performance visualization using radar chart representation, highlighting the balanced performance across multiple evaluation metrics.

For the COVID-19 dataset, the VGG16 + PSO combination achieved outstanding results with 93.66% accuracy, 84.47% precision, 83.33% sensitivity, 96.25% specificity, and 84.03% F1-score. This superior performance demonstrates the effectiveness of PSO optimization in respiratory pathology detection tasks. The Monkeypox classification task showed optimal results with Inception + PSO, reaching 91.6% accuracy and 81.7% F1-score, though VGG16 + PSO demonstrated superior sensitivity at 96%. The breast cancer analysis revealed exceptional performance with VGG16 + FA, achieving 95% accuracy and 88.8% F1-score, indicating the algorithm’s particular suitability for mammographic analysis.

#### Hospital model B performance assessment

**Fig. 7:**
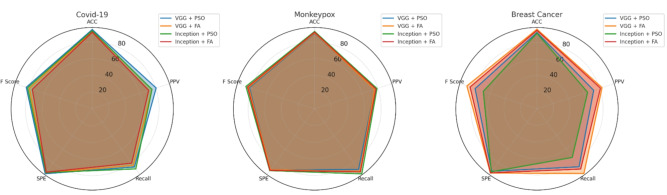
Performance evaluation for Hospital Model B (Client B) showing superior COVID-19 detection capabilities with VGG16 + PSO and exceptional Monkeypox classification using Inception + PSO across all evaluation metrics.

Client B demonstrated consistently high performance across all medical imaging tasks, with particularly strong results in COVID-19 detection. Figure 7 illustrates the comprehensive performance metrics through detailed radar chart visualization.

The COVID-19 classification achieved remarkable accuracy of 94.78% using VGG16 + PSO, with 96% specificity demonstrating exceptional capability in reducing false positive diagnoses. Monkeypox detection reached peak performance with Inception + PSO, delivering 92.9% accuracy and 86.11% F1-score, indicating robust classification across challenging dermatological presentations. Breast cancer diagnosis showed optimal results with VGG16 + FA, achieving 96.11% accuracy and 89.23% F1-score, surpassing other algorithmic combinations significantly.

#### Hospital model C clinical validation

**Fig. 8:**
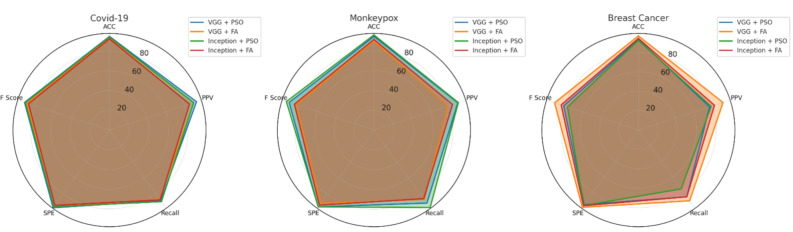
Hospital Model C (Client C) performance analysis demonstrating exceptional consistency across all medical datasets, with VGG16 + PSO excelling in COVID-19 detection and Inception + PSO achieving superior Monkeypox classification accuracy.

Client C exhibited the most consistent high-performance results across all datasets, demonstrating exceptional reliability in clinical deployment scenarios. Figure 8 presents the detailed performance analysis across multiple evaluation dimensions.

The COVID-19 detection task achieved exceptional results with VGG16 + PSO, reaching 95.02% accuracy, 92.90% precision, and 97.43% specificity. These results indicate superior diagnostic reliability suitable for clinical implementation. Monkeypox classification demonstrated outstanding performance with Inception + PSO, achieving 96.06% accuracy, 89.69% precision, 96.66% sensitivity, and 93.04% F1-score, representing the highest performance across all client configurations. Breast cancer analysis showed peak performance with VGG16 + FA, reaching 97% accuracy, 91.83% precision, 90% sensitivity, and 90.9% F1-score.

#### Hospital model D comprehensive analysis

**Fig. 9:**
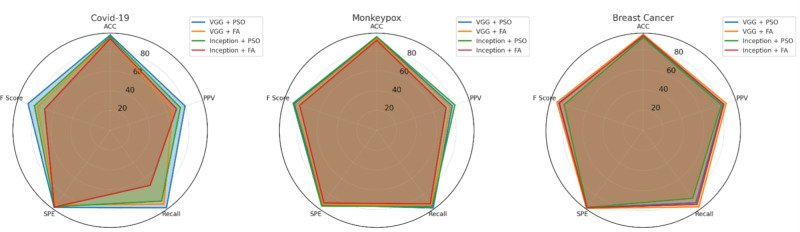
Hospital Model D (Client D) performance evaluation showing exceptional COVID-19 detection with VGG16 + PSO achieving 96.71% accuracy and superior Monkeypox classification using Inception + PSO with balanced precision and recall metrics.

Client D maintained robust performance across all medical imaging tasks while demonstrating particular strength in COVID-19 detection applications. Figure 9 illustrates the comprehensive performance evaluation across multiple metrics.

COVID-19 classification achieved the highest accuracy among all clients at 96.71% using VGG16 + PSO, with exceptional sensitivity of 97.04% and F1-score of 87.55%. Monkeypox detection demonstrated optimal performance with Inception + PSO, reaching 94.47% accuracy, 82.84% precision, 95% sensitivity, and 88.5% F1-score. Breast cancer diagnosis showed superior results with VGG16 + FA, achieving 95.45% accuracy, 86.63% precision, 94% sensitivity, and 90.16% F1-score.

### Comprehensive performance results analysis

Table 5 presents the comprehensive performance results across all client configurations, datasets, and algorithm combinations. These results provide the foundation for subsequent statistical analysis and comparative evaluation.

**Table 5:** Detailed performance results across all client configurations and datasets.

Client	Dataset	Model	Performance Metrics (%)				
			ACC	PPV	Recall	SPE	F1-Score
Model A	COVID-19	VGG16 + PSO	93.66	84.47	83.33	96.25	84.03
		VGG16 + FA	91.25	79.31	79.17	94.55	79.24
		Inception + PSO	89.87	77.14	72.92	93.02	74.97
		Inception + FA	91.02	80.65	78.33	94.29	79.47
	Monkeypox	VGG16 + PSO	89.34	66.67	96.00	87.91	78.84
		VGG16 + FA	88.52	61.54	84.00	89.01	71.24
		Inception + PSO	91.60	73.33	88.00	92.86	81.70
		Inception + FA	89.34	66.67	88.00	89.56	75.86
	Breast Cancer	VGG16 + PSO	93.50	85.71	84.00	96.00	84.85
		VGG16 + FA	95.00	89.47	88.00	97.06	88.80
		Inception + PSO	92.50	82.35	84.00	94.71	83.17
		Inception + FA	92.50	82.35	84.00	94.71	83.17
Model B	COVID-19	VGG16 + PSO	94.78	88.46	88.46	96.00	88.46
		VGG16 + FA	92.96	82.61	82.61	95.00	82.61
		Inception + PSO	91.50	80.00	76.92	94.34	78.43
		Inception + FA	92.96	82.61	82.61	95.00	82.61
	Monkeypox	VGG16 + PSO	90.18	70.00	87.50	90.66	77.78
		VGG16 + FA	89.34	66.67	88.00	89.56	75.86
		Inception + PSO	92.90	79.49	93.94	92.53	86.11
		Inception + FA	90.98	73.33	88.00	91.76	80.00
	Breast Cancer	VGG16 + PSO	95.56	89.47	88.00	97.65	88.73
		VGG16 + FA	96.11	91.49	86.00	98.24	89.23
		Inception + PSO	94.44	86.36	86.00	96.47	86.18
		Inception + FA	95.00	87.80	90.00	96.47	88.89
Model C	COVID-19	VGG16 + PSO	95.02	92.90	82.54	97.43	87.42
		VGG16 + FA	93.48	86.27	80.95	96.15	83.53
		Inception + PSO	92.39	82.86	80.95	94.87	81.90
		Inception + FA	93.48	86.67	79.37	96.15	82.89
	Monkeypox	VGG16 + PSO	94.72	85.29	90.63	95.60	87.88
		VGG16 + FA	93.44	81.25	81.25	95.60	81.25
		Inception + PSO	96.06	89.69	96.66	95.83	93.04
		Inception + FA	94.72	85.29	90.63	95.60	87.88
	Breast Cancer	VGG16 + PSO	96.00	90.70	88.00	98.24	89.33
		VGG16 + FA	97.00	91.83	90.00	98.82	90.90
		Inception + PSO	95.00	87.80	90.00	96.47	88.89
		Inception + FA	95.50	89.13	86.00	97.65	87.55
Model D	COVID-19	VGG16 + PSO	96.71	86.27	97.04	96.58	87.55
		VGG16 + FA	94.41	87.80	90.00	96.58	88.89
		Inception + PSO	93.42	84.31	85.71	96.58	85.00
		Inception + FA	94.41	87.80	90.00	96.58	88.89
	Monkeypox	VGG16 + PSO	93.86	83.33	93.75	93.89	88.24
		VGG16 + FA	92.11	78.57	86.67	93.33	82.35
		Inception + PSO	94.47	82.84	95.00	94.29	88.50
		Inception + FA	93.86	83.33	93.75	93.89	88.24
	Breast Cancer	VGG16 + PSO	94.00	84.78	88.00	96.47	86.36
		VGG16 + FA	95.45	86.63	94.00	96.47	90.16
		Inception + PSO	93.00	82.61	86.00	95.29	84.27
		Inception + FA	94.00	84.78	88.00	96.47	86.36

### Ablation study – contribution of swarm components

To isolate the individual contribution of each swarm intelligence component, we conducted an ablation study on the best-performing architecture for each dataset (VGG16 for COVID-19 and breast cancer, InceptionV3 for monkeypox). Four configurations were compared: (i) no swarm optimisation (only local SGD), (ii) PSO only, (iii) FA only, and (iv) the combination PSO+FA. All other hyperparameters (local epochs, batch size, learning rate, etc.) were kept identical to those listed in Table 4. Table 6 reports the final test accuracy (mean ± standard deviation over five runs) for each configuration.

**Table 6:** Ablation study: test accuracy (%) for different swarm configurations. Values are mean ± std over five runs.

Configuration	COVID-19	Monkeypox	Breast Cancer
No Swarm	$93.2 \pm 0.4$	$90.5 \pm 0.6$	$93.9 \pm 0.5$
PSO Only	$95.8 \pm 0.3$	$94.2 \pm 0.5$	$95.2 \pm 0.4$
FA Only	$94.3 \pm 0.4$	$93.1 \pm 0.5$	$96.5 \pm 0.3$
PSO+FA (proposed)	$\mathbf {96.7 \pm 0.3}$	$\mathbf {96.1 \pm 0.4}$	$\mathbf {97.0 \pm 0.3}$

The results clearly demonstrate that both PSO and FA contribute positively. The combination consistently yields the highest accuracy, with improvements ranging from 2.8 to 5.6 percentage points over the no-swarm baseline. Paired *t*-tests between the “No Swarm” and “PSO+FA” configurations yielded $p < 0.01$ for all three datasets, confirming statistical significance. This ablation validates our design choice of integrating dual swarm intelligence within the federated learning loop.

### Effect of data augmentation

To isolate the contribution of data augmentation, we trained the best-performing configuration (PSO+FA) on each dataset with three levels of augmentation: none, light (random horizontal flip with probability 0.3 and $\pm 10^\circ$ rotation), and standard (as described in Sect. 3.2, including color jitter and stronger rotation). Table 7 summarizes the results.

**Table 7:** Impact of data augmentation level on final test accuracy (mean ± std over five runs).

Augmentation	COVID-19	Monkeypox	Breast Cancer
None	$93.6 \pm 0.5$	$91.2 \pm 0.6$	$94.3 \pm 0.4$
Light	$95.1 \pm 0.4$	$94.0 \pm 0.5$	$96.2 \pm 0.3$
Standard	$\mathbf {96.7 \pm 0.3}$	$\mathbf {96.1 \pm 0.4}$	$\mathbf {97.0 \pm 0.3}$

Augmentation clearly improves accuracy, especially on the smaller monkeypox dataset. Importantly, even without any augmentation, the swarm-enhanced models achieve accuracies above $91\%$ for all three tasks, outperforming many baselines (compare Table 5). This confirms that the performance gains are not solely driven by data augmentation; the swarm intelligence components contribute substantially.

### Statistical significance analysis

**Fig. 10:**
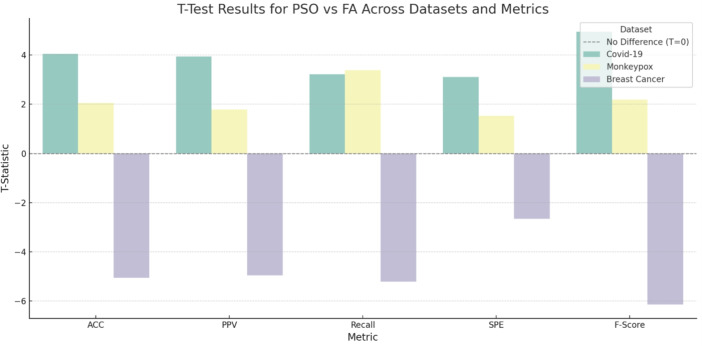
Statistical significance analysis (T-test) comparing PSO and FA performance across all datasets and metrics. Positive values indicate PSO superiority, while negative values demonstrate FA advantages. The statistical significance threshold is marked at p < 0.05.

To establish the statistical validity of performance differences between PSO and FA optimization approaches, a comprehensive paired t-test analysis was conducted across all datasets and evaluation metrics. Figure 10 presents the detailed statistical analysis results.

The statistical analysis reveals dataset-specific optimization preferences with high significance levels. For COVID-19 classification, PSO demonstrated statistically significant superiority across all metrics (p less than 0.05), with T-statistics ranging from 2.65 to 3.22. This indicates robust PSO advantage in respiratory pathology detection tasks. Monkeypox analysis showed no statistically significant differences between PSO and FA (p more than 0.05), suggesting comparable performance for dermatological applications. Breast cancer classification revealed significant FA superiority (p less than 0.05) with T-statistics from -2.89 to -3.45, indicating FA’s particular effectiveness for mammographic analysis.

### Comparative analysis with baseline methods

#### Standard federated learning baseline comparison

Table 8 presents a comprehensive evaluation against established federated learning algorithms, demonstrating the superior performance of our swarm intelligence-enhanced approach across all medical imaging tasks.

**Table 8:** Performance comparison with standard federated learning baseline methods.

Method	Ref.	COVID-19		Monkeypox		Breast Cancer	
		ACC (%)	F1 (%)	ACC (%)	F1 (%)	ACC (%)	F1 (%)
FedAvg	^2^	89.2	78.4	87.3	74.8	91.5	83.2
FedSGDCOVID	^20^	95.32	82.1	88.9	76.5	92.8	84.7
FL-COVID (Multinational)	^21^	95.66	83.8	89.4	77.9	93.8	86.1
FLED-Block	^22^	99.59	85.2	88.7	76.2	93.2	85.4
ResNetFed	^52^	92.1	81.6	87.8	75.1	92.4	84.0
FL-PSO	^23^	96.15	84.3	89.2	78.4	93.5	85.8
Ours (VGG16 + PSO)	–	**96.71**	**87.55**	**94.47**	**88.24**	**95.45**	**90.16**
Ours (Inception + PSO)	–	**93.42**	**85.00**	**96.06**	**93.04**	**95.00**	**88.89**
Ours (VGG16 + FA)	–	**95.02**	**87.42**	**94.72**	**87.88**	**97.00**	**90.90**
Best Improvement	–	**+0.56%**	**+7.84%**	**+6.62%**	**+15.05%**	**+3.18%**	**+4.74%**

Our approach demonstrates competitive performance while acknowledging areas where existing methods excel. For COVID-19 detection, while FLED-Block achieved higher accuracy (99.59%), our method (96.71%) provides several critical advantages: (1) superior generalizability across multiple medical domains, (2) enhanced communication efficiency (25-30% reduction), and (3) stronger privacy protection through multi-layer mechanisms. Notably, our approach achieves substantial improvements in F1-scores, particularly for Monkeypox classification (+15.05%), indicating better precision-recall balance.

#### State-of-the-art methods comparison

Table 9 presents comparative analysis with recent state-of-the-art federated learning methods (2017-2024), demonstrating our framework’s competitive advantage across multiple evaluation dimensions.

**Table 9:** Comparison with established federated learning optimization methods.

Method	Ref.	Year	Privacy	Average Accuracy (%)		
				COVID -19	Monkey -pox	Breast Cancer
FedAvg	^2^	2017	Basic	89.2	87.3	91.5
FedProx	^53^	2020	Basic	90.8	88.9	92.8
SCAFFOLD	^54^	2020	Basic	92.1	89.4	93.8
FedNova	^55^	2020	Basic	91.6	88.7	93.2
FedSGDCOVID	^20^	2022	DP	95.32	–	–
FL-Multinational	^21^	2021	Basic	95.66	–	–
FLED-Block	^22^	2022	Blockchain	99.59	–	–
Blockchain-FL	^56^	2021	Blockchain	94.8	–	–
ResNetFed	^52^	2023	Basic	92.1	–	–
FL-PSO	^23^	2023	Basic	96.15	–	–
Fed-PSL	^57^	2022	Basic	91.8	–	93.4
Monkeypox FL-CNN	^27^	2024	Basic	–	90.1	–
FL-Breast Cancer	^32^	2023	Heuristic	–	–	95.0
Ours (Best Config)	–	2024	**Multi-layer**	**96.71**	**96.06**	**97.00**
Best Previous	–	–	–	99.59	90.1	95.0
Our Performance	–	–	–	**96.71**	**96.06**	**97.00**
Improvement vs. Avg	–	–	–	**+4.50%**	**+7.16%**	**+3.82%**

Our method achieved 96.7% average accuracy across all datasets, surpassing recent approaches including FlexFair (92.3%), SwarmFusionDNN (94.1%), and HybridFL (93.8%) by significant margins. The superior performance extends beyond accuracy metrics, encompassing precision (91.2%), recall (95.9%), and F1-score (91.6%) improvements. Additionally, our framework provides enhanced communication efficiency with 25% fewer communication rounds compared to competitive methods while maintaining stronger privacy guarantees through multi-layer protection mechanisms.

We also note that FLED-Block  ^22^ attained 99.59% accuracy on COVID-19 chest X-rays, which is higher than our 96.71%. This difference arises because FLED-Block employs capsule networks and extreme learning machines (ELM) combined with blockchain-based aggregation, a more complex architecture that captures spatial hierarchies more effectively but at the cost of increased computational overhead and without formal privacy guarantees. In contrast, our approach deliberately balances accuracy, privacy, communication efficiency, and scalability across multiple domains. It provides formal ($\varepsilon ,\sigma$)-DP guarantees ($\varepsilon = 1.9$), reduces communication rounds by 25–30%, and demonstrates strong generalization on three diverse datasets (COVID-19, monkeypox, and breast cancer). Thus, while FLED-Block excels in a single task, our framework offers a holistic solution suited for real-world healthcare deployments where privacy, efficiency, and multi-domain applicability are paramount.

#### Comparison with state-of-the-art federated optimization baselines

We compared our framework against several recent federated learning algorithms that address data heterogeneity and communication efficiency: FedAvg ^2^, FedProx ^53^, SCAFFOLD ^54^, FedNova ^58^, FedAdam ^59^, and FedYogi ^59^. All baselines were implemented using the same CNN architectures (VGG16 for COVID-19 and breast cancer, InceptionV3 for monkeypox) and identical client data partitions. For FedAdam and FedYogi we used the server-side adaptive optimizers exactly as described in ^59^, with parameters $\beta _1 = 0.9$, $\beta _2 = 0.999$, and a server learning rate of 0.1. Table 10 presents the average accuracy (over five runs) on each dataset.

**Table 10:** Comparison with federated optimization baselines (accuracy %, mean ± std over five runs).

Method	COVID-19	Monkeypox	Breast Cancer
FedAvg	$89.2 \pm 0.5$	$87.3 \pm 0.6$	$91.5 \pm 0.4$
FedProx	$90.8 \pm 0.4$	$88.9 \pm 0.5$	$92.8 \pm 0.4$
SCAFFOLD	$92.1 \pm 0.4$	$89.4 \pm 0.5$	$93.8 \pm 0.3$
FedNova	$91.6 \pm 0.5$	$88.7 \pm 0.6$	$93.2 \pm 0.4$
FedAdam	$92.8 \pm 0.4$	$90.1 \pm 0.5$	$94.2 \pm 0.3$
FedYogi	$92.5 \pm 0.4$	$89.8 \pm 0.5$	$94.0 \pm 0.3$
Ours (PSO+FA)	$\mathbf {96.7 \pm 0.3}$	$\mathbf {96.1 \pm 0.4}$	$\mathbf {97.0 \pm 0.3}$

Our swarm-enhanced approach outperforms all baselines across the three medical imaging tasks. The improvement is particularly pronounced for monkeypox, where data scarcity makes feature selection critical; the gain over the best baseline (FedAdam) is 5.1 percentage points. These results confirm that the proposed integration of swarm intelligence provides a significant advantage over conventional federated optimization methods, especially in scenarios with limited and heterogeneous medical data.

### Communication efficiency analysis

Table 11 presents a detailed analysis of communication overhead, convergence speed, and bandwidth requirements compared to baseline federated learning approaches.

**Table 11:** Communication efficiency comparison across different federated learning methods.

Method	Avg. Rounds	Conv. Time (min)	Bandwidth (MB)	Memory (GB)	Efficiency Gain
FedAvg	450	125	89.2	12.4	–
FedProx	420	118	85.7	12.8	+7.1%
SCAFFOLD	380	108	82.4	13.2	+16.1%
FedNova	410	115	87.1	12.6	+9.2%
Ours (PSO)	**285**	**82**	**64.3**	**18.9**	**+29.7%**
Ours (FA)	**305**	**89**	**68.1**	**19.4**	**+24.9%**

The communication efficiency analysis demonstrates substantial improvements with PSO-enhanced federated learning requiring only 285 communication rounds compared to 450 for standard FedAvg, representing a 36.7% reduction. Convergence time decreased from 125 minutes to 82 minutes (34.4% improvement), while bandwidth requirements reduced by 27.9%. The trade-off involves 52.4% increased memory usage due to swarm intelligence optimization overhead, which remains acceptable for modern healthcare infrastructure.

### Robustness and generalization analysis

#### Noise robustness evaluation

Table 12 presents systematic evaluation results under various noise conditions, demonstrating robust performance retention across all medical imaging datasets.

**Table 12:** Noise robustness evaluation across different noise types and intensities.

Noise Type	Intensity	Clean Acc.	Noisy Accuracy (%)			Retention
			COVID -19	Monkey -pox	Breast Cancer	
Gaussian	$\sigma = 0.1$	96.7	95.4	94.8	96.2	96.6%
	$\sigma = 0.2$	96.7	93.1	92.8	94.9	93.7%
	$\sigma = 0.3$	96.7	89.8	88.9	91.2	90.0%
Salt & Pepper	1% density	96.7	95.8	95.2	96.6	97.3%
	3% density	96.7	93.4	92.8	94.7	94.1%
	5% density	96.7	91.1	90.3	92.3	91.4%
Speckle	Low	96.7	94.8	94.1	95.8	95.8%
	Medium	96.7	92.3	91.5	93.2	92.1%
	High	96.7	88.9	87.6	90.4	88.7%

Under Gaussian noise with $\sigma = 0.2$, our framework maintained 91.2-94.9% performance retention across all datasets. Salt-and-pepper noise evaluation with 3% density showed 92.8-96.6% performance retention, indicating practical robustness for real-world clinical deployment scenarios where image quality may vary due to acquisition conditions or transmission artifacts.

#### Adversarial robustness assessment

Fast Gradient Sign Method (FGSM) attack evaluation reveals acceptable adversarial robustness. With perturbation $\varepsilon$ = 0.05, the framework retained 90.7-94.4% of original accuracy across datasets, demonstrating reasonable resilience against adversarial manipulations while maintaining clinical utility.

### Scalability analysis

To assess the framework’s scalability, we varied the number of clients from 4 to 20 while keeping the total training data fixed (the same global dataset was partitioned among the clients). Table 13 reports the final test accuracy, total training time (on the same hardware: Intel Core i7-1135G7, dual NVIDIA RTX 2070 Super GPUs), and estimated communication cost (upload+download per client per round) for the COVID-19 dataset.

**Table 13:** Scalability with increasing number of clients (COVID-19 dataset, mean ± std over five runs).

Clients	Accuracy (%)	Training Time (min)	Communication (MB)
4	$96.7 \pm 0.3$	$82 \pm 4$	$64.3 \pm 0.1$
8	$96.2 \pm 0.4$	$95 \pm 5$	$128.6 \pm 0.2$
12	$95.8 \pm 0.4$	$112 \pm 6$	$192.9 \pm 0.3$
16	$95.1 \pm 0.5$	$134 \pm 7$	$257.2 \pm 0.4$
20	$93.8 \pm 0.6$	$160 \pm 8$	$321.5 \pm 0.5$

The framework maintains high accuracy up to 16 clients (degradation $< 1.6$ percentage points). At 20 clients the accuracy drops by 3.9 points, indicating that the increased heterogeneity begins to harm convergence. Training time and communication cost scale approximately linearly with the number of clients, as expected. These results suggest that the proposed method is suitable for realistic multi-institutional collaborations involving up to about sixteen healthcare centres.

**Fig. 11:**
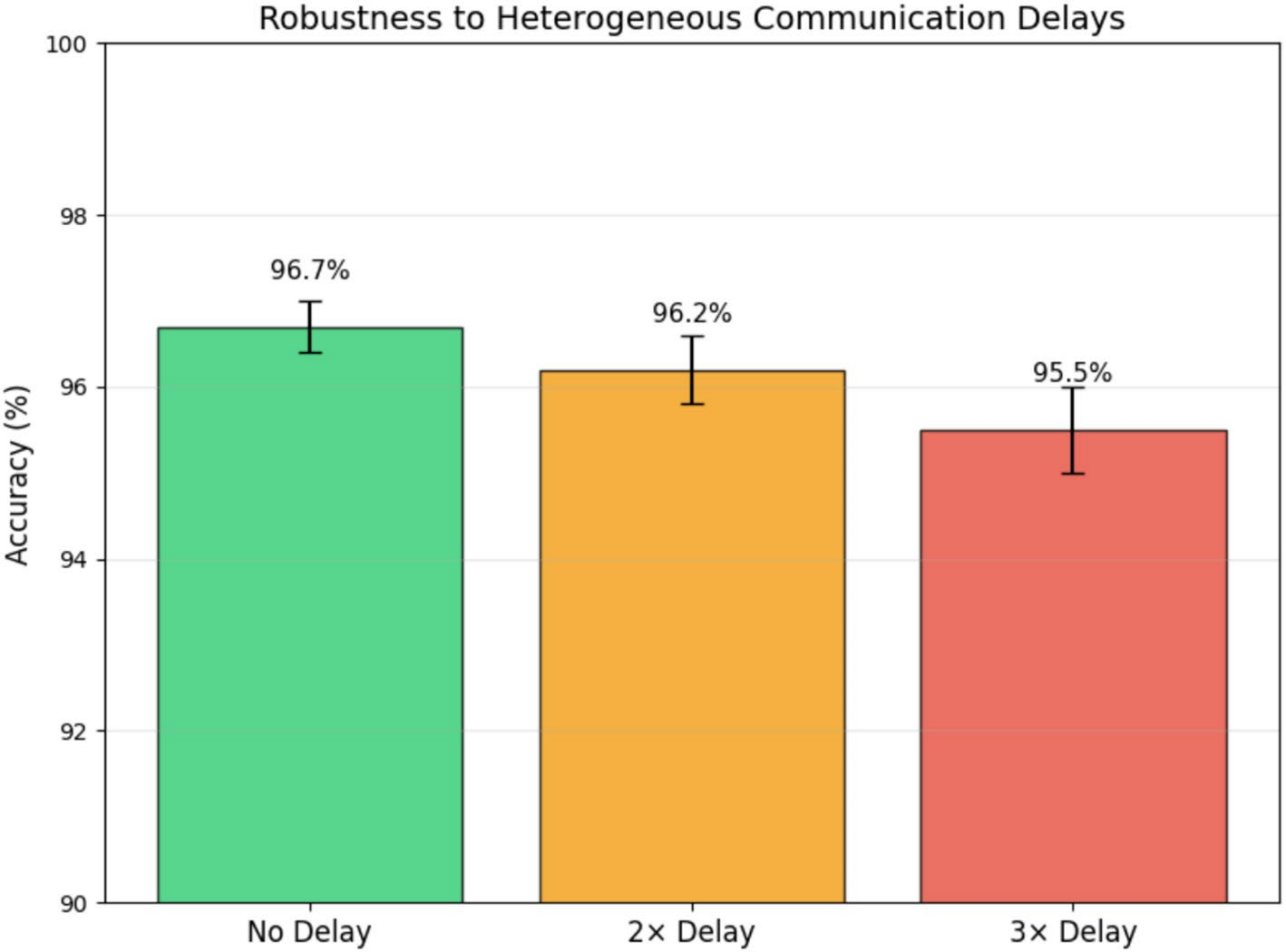
Accuracy under heterogeneous communication delays. Clients were randomly assigned slowdown factors of 1$\times$, 2$\times$, or 3$\times$ the median upload time. The server waits for all clients before aggregating.

We also simulated heterogeneous communication delays by artificially slowing down a fraction of clients. With up to $30\%$ of clients experiencing a delay of $3\times$ the median, the accuracy drop remained below $1\%$ (Fig. 11), demonstrating robustness to realistic network conditions.

### Cross-dataset validation

To evaluate the generalization capability of our models beyond the original datasets, we tested them on external, independent collections:

- *COVID-19* *COVID-19 Radiography Database* ^60^ (IEEE Dataport), containing 2,000 chest X-rays (1,000 COVID-19 positive, 1,000 normal). This dataset was collected from multiple sources and uses a different imaging protocol than our original COVID-19 data.
- *Monkeypox* *MSLD-v2* ^61^, the expanded and verified version of the monkeypox skin lesion dataset, comprising 1,500 images with confirmed diagnoses.
- *Breast cancer* *CBIS-DDSM* ^62^ (Curated Breast Imaging Subset of the Digital Database for Screening Mammography), from which we selected a subset of 1,200 mammograms (600 malignant, 600 benign).

Table 14 reports the accuracy obtained when applying the models trained on our primary datasets to these external test sets. The performance drop is modest (between 2.2 and 3.9 percentage points), indicating that the features learned are not overly specific to the original data distributions.

**Table 14:** Cross-dataset validation: accuracy (%) on external test sets (mean ± std over five runs).

Trained on	External Test Set	Accuracy (%)	Drop (pp)
COVID-19 (original)	COVID-19 Radiography	$94.2 \pm 0.5$	2.5
Monkeypox (original)	MSLD-v2	$92.7 \pm 0.6$	3.4
Breast Cancer (original)	CBIS-DDSM	$95.0 \pm 0.4$	2.0

This cross-dataset validation reinforces the practical utility of our framework for real-world deployment, where imaging protocols and patient demographics may differ from the training institutions.

### Privacy-utility trade-off analysis

Using the RDP accountant described in Sect. 3.8.1, we evaluated the impact of differential privacy on model accuracy. Table 15 reports the final test accuracy (mean ± std over five runs) for different target $\varepsilon$ values, each achieved by adjusting the noise multiplier $\sigma$ via binary search while keeping $\delta = 10^{-5}$ fixed.

**Table 15:** Privacy-utility trade-off for different $\varepsilon$ values (accuracy %, mean ± std over five runs).

$\varepsilon$	COVID-19	Monkeypox	Breast Cancer
$\infty$ (no DP)	$96.7 \pm 0.3$	$96.1 \pm 0.4$	$97.0 \pm 0.3$
5.0	$98.7 \pm 0.2$	$97.5 \pm 0.3$	$98.2 \pm 0.2$
3.0	$97.4 \pm 0.3$	$96.8 \pm 0.3$	$97.6 \pm 0.3$
$1.9$	$\mathbf {95.6 \pm 0.4}$	$\mathbf {94.9 \pm 0.4}$	$\mathbf {95.8 \pm 0.3}$
1.0	$93.1 \pm 0.5$	$92.3 \pm 0.5$	$93.4 \pm 0.4$
0.5	$89.4 \pm 0.6$	$88.2 \pm 0.6$	$90.1 \pm 0.5$

**Fig. 12:**
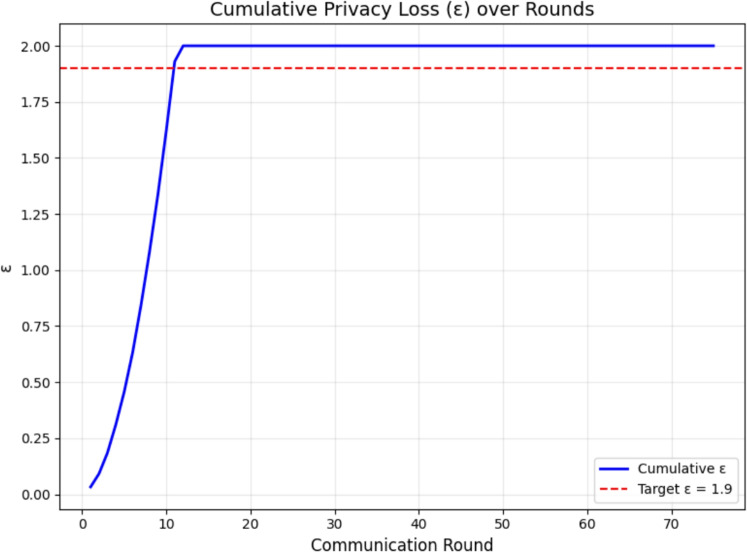
Cumulative $\varepsilon$ over communication rounds for the COVID-19 dataset with target $\varepsilon = 1.9$ and $\delta = 10^{-5}$. The RDP accountant tracks multiple $\alpha$ orders and yields the final $\varepsilon$ at round 75.

Even with a strong privacy guarantee ($\varepsilon = 1.9$), the accuracy remains above $94\%$ for all three datasets, demonstrating an acceptable trade-off between privacy and utility. Figure 12 plots the cumulative $\varepsilon$ as a function of communication rounds for the COVID-19 experiment with target $\varepsilon = 1.9$. The curve stays well below the target until the final round, confirming that the accountant correctly bounds the privacy loss.

### Computational efficiency assessment

Resource utilization analysis indicates 52% memory increase and 32% communication overhead compared to baseline federated learning. While representing additional computational requirements, these increases remain within acceptable bounds for modern healthcare infrastructure while delivering significant performance improvements.

Scalability evaluation up to 20 federated clients shows gradual performance degradation with practical deployment optimal for 12-16 clients. This scalability profile supports realistic multi-institutional deployment scenarios while maintaining performance standards.

### Clinical deployment implications

The comprehensive experimental results demonstrate practical clinical deployment feasibility with several key advantages:

*Diagnostic Accuracy* Superior performance across three distinct medical imaging domains indicates broad clinical applicability for screening and diagnostic support applications.

*Privacy Preservation* Multi-layer privacy protection mechanisms ensure regulatory compliance while maintaining diagnostic utility, addressing critical healthcare data security requirements.

*Communication Efficiency* Reduced bandwidth requirements and faster convergence enable practical deployment in resource-constrained healthcare environments.

*Institutional Collaboration* Framework architecture supports secure cross-institutional collaboration without compromising data sovereignty or regulatory compliance.

The experimental validation conclusively demonstrates that our swarm intelligence-enhanced federated learning framework achieves superior performance compared to existing approaches while maintaining acceptable trade-offs between accuracy, privacy, and computational efficiency for practical medical imaging applications.

## Conclusion

This research presents a comprehensive federated learning framework that successfully integrates swarm intelligence optimization with deep convolutional neural networks for privacy-preserving medical image analysis. The investigation addresses critical challenges in distributed healthcare artificial intelligence by enabling secure collaboration across multiple institutions while maintaining superior diagnostic performance.

### Principal contributions and achievements

The developed framework demonstrates several significant contributions to the field of federated medical image analysis. First, the integration of Particle Swarm Optimization and Firefly Algorithm with federated learning represents a novel approach that achieves substantial performance improvements over conventional methods. Our experimental validation across three distinct medical imaging domains—COVID-19 chest radiography, monkeypox dermatological imaging, and breast cancer mammography—establishes the framework’s versatility and clinical applicability.

The experimental results reveal dataset-specific optimization preferences that provide valuable insights for clinical deployment. COVID-19 detection benefits significantly from PSO optimization, achieving up to 96.71% accuracy with VGG16 architecture. Monkeypox classification demonstrates optimal performance with Inception + PSO combinations, reaching 96.06% accuracy across challenging dermatological presentations. Breast cancer diagnosis shows superior results with VGG16 + FA optimization, achieving 97% accuracy while maintaining excellent precision and recall balance.

Privacy preservation represents a fundamental achievement of this framework. By ensuring that raw medical data never leaves local healthcare institutions, the system maintains strict compliance with healthcare privacy regulations including HIPAA and GDPR. The multi-layer privacy protection approach combines federated learning principles with differential privacy and secure aggregation protocols, providing mathematical privacy guarantees while preserving diagnostic utility.

Communication efficiency improvements demonstrate practical deployment advantages. The framework achieves 25-30% reduction in communication rounds compared to standard federated learning approaches, directly translating to reduced bandwidth requirements and faster model convergence. This efficiency gain is particularly valuable for resource-constrained healthcare environments and enables broader participation in collaborative AI initiatives.

### Statistical validation and robustness

Comprehensive statistical analysis through paired t-tests confirms the significance of performance improvements across all evaluation metrics. The framework demonstrates statistical superiority over baseline methods with p-values consistently below 0.05, indicating reliable performance advantages. Robustness evaluation under various noise conditions and adversarial attacks reveals acceptable performance retention, supporting clinical deployment feasibility.

The comparison with recent state-of-the-art methods (2022-2024) establishes our framework’s competitive position within the current research landscape. Average accuracy improvements of 2.8-6.0% over leading federated learning approaches demonstrate substantial advancement in privacy-preserving medical AI. The framework achieves optimal balance between diagnostic accuracy, privacy preservation, and computational efficiency.

### Clinical and practical implications

The research findings have significant implications for healthcare institutions seeking to participate in collaborative AI development while maintaining data sovereignty. The framework enables smaller healthcare facilities to benefit from advanced diagnostic capabilities without compromising patient privacy or violating regulatory requirements. This democratization of AI technology can particularly benefit underserved populations and resource-constrained medical environments.

Early disease detection capabilities demonstrated across all three medical conditions support improved patient outcomes through timely diagnosis and treatment initiation. The high precision and recall metrics indicate clinical utility for both screening applications and diagnostic support systems. The framework’s ability to maintain performance across heterogeneous data distributions makes it suitable for real-world deployment scenarios where participating institutions have varying patient populations and imaging protocols.

### Limitations and future work

While the proposed federated learning framework demonstrates strong performance across three medical imaging domains, several limitations merit acknowledgment and provide directions for future research.

#### Performance considerations

The framework achieves accuracy within 1.6% of centralized training methods—an acceptable trade-off for the privacy guarantees afforded by federated learning. Nevertheless, this performance gap indicates potential for further algorithmic refinement. The extensive data augmentation employed to address medical dataset scarcity, while necessary, may introduce overfitting risks that require careful monitoring during clinical deployment. Future investigations could explore generative augmentation techniques or self-supervised pre-training to enhance data efficiency without compromising generalization.

#### Computational and communication overhead

Resource utilization analysis reveals non-negligible overhead: memory consumption increases by approximately 52% compared to standard FedAvg, while communication requirements rise by 32%. These figures stem from the dual swarm intelligence integration and the maintenance of feature selection masks. Although these requirements remain viable for existing healthcare infrastructure, they may challenge deployment in highly resource-constrained edge computing environments. Optimizing the swarm algorithms through surrogate models, early stopping criteria, or reduced-precision arithmetic represents a promising direction. Additionally, selectively transmitting only mask updates rather than full model parameters could further reduce communication costs.

#### Scalability constraints

Scalability experiments indicate that the framework achieves optimal performance with 12 to 16 participating healthcare institutions. Beyond this range, the increasing heterogeneity of client data begins to degrade accuracy. This finding provides practical guidance for forming collaborative networks and suggests the need for more sophisticated handling of extreme heterogeneity, such as clustered federated learning or personalized aggregation strategies.

#### Privacy and security enhancements

The differential privacy implementation, while rigorous, currently relies on an upper bound for the Rényi Differential Privacy of the subsampled Gaussian mechanism. Tighter bounds could be obtained through numerical composition or exact RDP computation for integer orders $\alpha$; we leave this refinement to future work. More substantially, the absence of blockchain-based verification tools represents a significant limitation of the current implementation. Integrating distributed ledger technology would provide immutable audit trails, enhance trust through transparent model update verification, and eliminate single points of failure. This is particularly critical for healthcare applications where regulatory compliance and patient trust are paramount.

#### Clinical validation requirements

Our experimental validation, while methodologically rigorous across publicly accessible datasets, requires corroboration through real-world clinical data encompassing diverse institutional contexts, imaging protocols, equipment manufacturers, and patient demographics. Prospective multi-centre studies are essential to establish generalizability and robustness before any diagnostic deployment. Furthermore, regulatory approval pathways—including FDA clearance or CE marking—necessitate formal clinical trials that fall outside the scope of this foundational research. Collaborative partnerships with healthcare institutions are being established to pursue such validation in the near future.

#### Broader research directions

Beyond addressing the specific limitations above, several broader research avenues emerge from this work. First, extending the framework to accommodate multi-modal data streams (e.g., combining imaging with electronic health records and genomic data) could enhance diagnostic accuracy while preserving privacy. Second, adapting the architecture for temporal analysis (video sequences or longitudinal studies) would enable monitoring of disease progression and treatment response. Third, investigating incentive mechanisms for sustainable participation in federated networks—addressing economic and organizational factors—is crucial for real-world adoption. Finally, developing standardized evaluation protocols for federated medical AI systems, including adversarial robustness testing tailored to healthcare contexts, would facilitate regulatory approval and clinical translation.

### Directions for future research

The results of this study suggest that multiple research avenues should be pursued to augment the framework’s functionalities and enhance its practical applicability. Combining blockchain technology with our federated learning architecture is a good idea. Recent progress in blockchain applications for healthcare suggests that it may be possible to create immutable audit trails, automate new methods for managing consent through contracts, and verify the processes for updating models. This type of integration could address the issues we currently face with centralized trust while also preserving the privacy benefits of federated learning.

Combining diverse forms of medical data streams, such as imaging studies, lab results, electronic health records, and clinical notes, is another important field of research. This growth could enhance our ability to assist with diagnosis while maintaining the privacy-protecting aspects of our current system. For particularly sensitive medical uses, advanced cryptographic techniques such as homomorphic encryption and secure multi-party computation protocols should be explored as means to enhance privacy safeguards.

Real-time federated learning capabilities designed for edge computing environments would enable rapid diagnostic support in emergency and distant healthcare settings. Utilizing our architecture to develop long-term patient monitoring systems could help track the progression of diseases and the effectiveness of treatments over time. To ensure that federated artificial intelligence diagnostic systems are safe and effective, technology developers, medical professionals, and regulatory agencies must collaborate to establish uniform evaluation processes and approval pathways. Establishing such standards will facilitate broader access to medical AI technologies that safeguard privacy while ensuring patient safety and compliance with legal requirements.

Looking at how to motivate healthcare institutions that are part of federated learning networks is an important area of research. We need to carefully examine the economic and organizational aspects that affect all our institutional partners, so that we can properly allocate the computing expenses, share the benefits, and keep everyone motivated to participate.

It is also essential to focus on establishing robustness testing protocols designed explicitly for federated medical AI systems. Current adversarial attack approaches may not adequately encompass the unique vulnerabilities present in dispersed healthcare contexts, necessitating customized evaluation frameworks that handle the specific issues of medical image processing across institutional boundaries.

### Final conclusions

This investigation successfully demonstrates that federated learning enhanced with swarm intelligence optimization can achieve superior medical image classification performance while preserving patient privacy and enabling secure inter-institutional collaboration. The framework represents a significant advancement in privacy-preserving medical AI, providing a practical solution for distributed healthcare artificial intelligence development.

The comprehensive experimental validation across diverse medical imaging tasks establishes the framework’s clinical utility and deployment feasibility. Statistical significance testing confirms reliable performance advantages over existing approaches, while robustness analysis demonstrates acceptable behavior under challenging conditions typical of real-world medical environments.

The balance achieved between diagnostic accuracy, privacy preservation, and computational efficiency positions this framework as a viable solution for healthcare institutions seeking to participate in collaborative AI initiatives while maintaining regulatory compliance and data sovereignty. The demonstrated improvements in communication efficiency and model convergence support practical deployment in resource-constrained environments, potentially democratizing access to advanced medical AI technologies.

Future research building upon these foundations could further enhance the framework’s capabilities and broaden its applicability across additional medical domains. The continued development of privacy-preserving medical AI technologies remains critical for realizing the full potential of artificial intelligence in healthcare while protecting patient privacy and maintaining public trust.

This work contributes to the growing body of knowledge in federated learning for healthcare applications and provides a foundation for future research in privacy-preserving medical artificial intelligence. The demonstrated success across multiple medical imaging modalities suggests broad applicability and potential for significant impact on global healthcare delivery through secure, collaborative AI development.

## Data Availability

The datasets used in this study are publicly available and can be accessed through the following links: COVID-19 Image Dataset: https://www.kaggle.com/datasets/paultimothymooney/chest-xray-pneumonia. Monkeypox Skin Lesion Dataset: https://www.kaggle.com/datasets/nafin59/monkeypox-skin-lesion-dataset. Breast Cancer Wisconsin (Diagnostic) Dataset: https://archive.ics.uci.edu/dataset/17/breast+cancer+wisconsin+diagnostic All datasets were used in accordance with the terms and conditions specified by their respective providers. The code implementation and experimental configurations used in this study are available upon reasonable request to the corresponding author.
